# Serial millisecond crystallography of membrane and soluble protein microcrystals using synchrotron radiation

**DOI:** 10.1107/S205225251700570X

**Published:** 2017-05-24

**Authors:** Jose M. Martin-Garcia, Chelsie E. Conrad, Garrett Nelson, Natasha Stander, Nadia A. Zatsepin, James Zook, Lan Zhu, James Geiger, Eugene Chun, David Kissick, Mark C. Hilgart, Craig Ogata, Andrii Ishchenko, Nirupa Nagaratnam, Shatabdi Roy-Chowdhury, Jesse Coe, Ganesh Subramanian, Alexander Schaffer, Daniel James, Gihan Ketwala, Nagarajan Venugopalan, Shenglan Xu, Stephen Corcoran, Dale Ferguson, Uwe Weierstall, John C. H. Spence, Vadim Cherezov, Petra Fromme, Robert F. Fischetti, Wei Liu

**Affiliations:** a School of Molecular Sciences and Biodesign Center for Applied Structural Discovery, Biodesign Institute, Arizona State University, Tempe, AZ 85287, USA; b Structural Biophysics Laboratory, National Cancer Institute, Frederick, MD 21702, USA; c Department of Physics, Arizona State University, PO Box 871504, Tempe, AZ 85287, USA; d Advanced Photon Source, Argonne National Laboratory, 9700 South Cass Avenue, Lemont, IL 60439, USA; e Department of Chemistry, Bridge Institute, University of Southern California, 3430 South Vermont Avenue, MC 3303, Los Angeles, CA 90089, USA; f Paul Scherrer Institute, 5232 Villigen, Switzerland

**Keywords:** serial millisecond crystallography, synchrotron radiation, Advanced Photon Source, high-viscosity injector

## Abstract

Crystal structure determination of biological macromolecules using the novel technique of serial femtosecond crystallography (SFX) is severely limited by the scarcity of X-ray free-electron laser (XFEL) sources. However, recent and future upgrades render microfocus beamlines at synchrotron-radiation sources suitable for room-temperature serial crystallography data collection also. Owing to the longer exposure times that are needed at synchrotrons, serial data collection is termed serial millisecond crystallography (SMX). As a result, the number of SMX experiments is growing rapidly, with a dozen experiments reported so far. Here, the first high-viscosity injector-based SMX experiments carried out at a US synchrotron source, the Advanced Photon Source (APS), are reported. Microcrystals (5–20 µm) of a wide variety of proteins, including lysozyme, thaumatin, phycocyanin, the human A_2A_ adenosine receptor (A_2A_AR), the soluble fragment of the membrane lipoprotein Flpp3 and proteinase K, were screened. Crystals suspended in lipidic cubic phase (LCP) or a high-molecular-weight poly(ethylene oxide) (PEO; molecular weight 8 000 000) were delivered to the beam using a high-viscosity injector. In-house data-reduction (hit-finding) software developed at APS as well as the SFX data-reduction and analysis software suites *Cheetah* and *CrystFEL* enabled efficient on-site SMX data monitoring, reduction and processing. Complete data sets were collected for A_2A_AR, phycocyanin, Flpp3, proteinase K and lysozyme, and the structures of A_2A_AR, phycocyanin, proteinase K and lysozyme were determined at 3.2, 3.1, 2.65 and 2.05 Å resolution, respectively. The data demonstrate the feasibility of serial millisecond crystallography from 5–20 µm crystals using a high-viscosity injector at APS. The resolution of the crystal structures obtained in this study was dictated by the current flux density and crystal size, but upcoming developments in beamline optics and the planned APS-U upgrade will increase the intensity by two orders of magnitude. These developments will enable structure determination from smaller and/or weakly diffracting microcrystals.

## Abstract 2

In this proof-of-principle study, the feasibility of structure determination of several proteins using serial millisecond crystallography (SMX) has been evaluated. The first high-viscosity injector-based SMX experiments carried out at a US synchrotron source, the Advanced Photon Source (APS), are reported.

## 1. Introduction   

Despite the deposition of over 115 000 crystal structures of biological macromolecules in the Protein Data Bank (PDB; http://www.rcsb.org), traditional techniques of macromolecular X-ray crystallography have always suffered from two major bottlenecks: the production of large, well diffracting crystals and radiation damage. The former has been especially prevalent in the structure determination of membrane proteins and large complexes, which can require months or even years of devoted effort to optimize conditions for the growth of crystals of sufficient quality, while the latter has hindered progress in the structural determination of radiation-sensitive proteins. For decades, the effect of radiation damage in macromolecular crystallography has been addressed by cryocooling crystals (Low *et al.*, 1966 ▸; Macchi, 2012 ▸; Pflugrath, 2004 ▸), thus extending their lifetime during X-ray beam exposure. However, third-generation synchrotrons produce such intense microfocus X-ray beams that rapid photo-damage can rapidly accumulate even under cryogenic conditions (Ravelli & Garman, 2006 ▸). Both the crystal-size and radiation-damage limitations have now been overcome by X-ray free-electron lasers (XFELs), the brightest X-ray sources, which are capable of producing extremely intense femtosecond X-ray pulses. Taking advantage of these unique properties, a new method of crystallography has emerged, termed serial femtosecond crystallography (SFX; Chapman *et al.*, 2011 ▸). In SFX, hundreds of thousands of nanocrystals and microcrystals suspended in their mother liquor or in a viscous carrier are delivered to the X-ray beam in a jet. The duration of exposure per crystal is so short (typically 40 fs) that diffraction can be recorded by a fast, integrating detector before the beam destroys the crystal, thereby outrunning radiation damage (Chapman *et al.*, 2011 ▸; Neutze *et al.*, 2000 ▸). The high intensity of the XFEL pulses used in SFX eliminates the need to grow large crystals. Inspired by this novel method­ology, a number of innovations such as nanocrystallization and microcrystallization techniques (Kupitz *et al.*, 2014 ▸; Liu *et al.*, 2014 ▸; Redecke *et al.*, 2013 ▸), serial sample-delivery methods (Ayvazyan *et al.*, 2006 ▸; Feld *et al.*, 2015 ▸; Huang *et al.*, 2015 ▸; Sierra *et al.*, 2012 ▸; Weierstall *et al.*, 2012 ▸, 2014 ▸), fast-readout detector technology (*e.g.* CSPAD) and rapid, high-throughput data-reduction and data-processing software have been developed (Barty *et al.*, 2014 ▸; Kirian *et al.*, 2010 ▸, 2011 ▸; Sauter, 2015 ▸; Sawaya *et al.*, 2014 ▸; White *et al.*, 2012 ▸, 2013 ▸, 2016 ▸). Consequently, numerous SFX crystal structures of membrane and soluble proteins have now been solved and deposited in the PDB (for a review, see Martin-Garcia *et al.*, 2016 ▸), showing the increasing utility of this technology to structural biologists.

Despite the advantages mentioned above, the use of SFX is severely limited by the scarcity of XFEL facilities. Currently, only two XFELs are operational in the world: the Linac Coherent Light Source (LCLS) at the SLAC National Accelerator Laboratory, Stanford, California, USA and the SPring-8 Angstrom Compact Free-Electron Laser (SACLA) in Japan. Unlike synchrotrons, the circular design of which allows the placement of many beamlines around the storage ring, XFELs are limited to only a few beamlines, only one of which can collect diffraction data at any given time, owing to their linear design. Even though several XFEL sources are currently under construction (PAL in South Korea, SwissFEL in Switzerland and the European XFEL in Germany) and are expected to enter the commissioning phase in 2017, the total number of XFELs will still be too low to meet the growing needs of the structural biology community compared with the numerous synchrotron facilities currently available worldwide (over 30 synchrotrons; http://www.esrf.eu/UsersAndScience/Links/Synchrotrons). To overcome this limitation, the structural biology community has begun to adopt the serial crystallography approach at the microfocus beamlines of third-generation synchrotron-radiation sources, which produce bright micrometre-sized X-ray beams and are equipped with fast-readout detectors (*e.g.* PILATUS and EIGER). However, since the brilliance of synchrotrons is not as high as that of XFELs, at least a millisecond exposure is required to produce sufficiently strong diffraction. Here, the microcrystals are delivered either loaded into fixed-target devices or continuously in single file across the beam in a viscous host medium. Because a goniometer is not used, a sufficiently viscous medium is important to prevent significant rotation of the crystals during exposure. The change in diffraction conditions is therefore negligible if the rotational diffusion time of the crystal in the medium is much longer than the exposure time. Thus, this method has been named serial millisecond crystallography (SMX). Over the last three years, several SMX trials have been published (Botha *et al.*, 2015 ▸; Coquelle *et al.*, 2015 ▸; Gati *et al.*, 2014 ▸; Hasegawa *et al.*, 2016 ▸; Heymann *et al.*, 2014 ▸; Huang *et al.*, 2015 ▸, 2016 ▸; Murray *et al.*, 2015 ▸; Nogly *et al.*, 2015 ▸; Stellato *et al.*, 2014 ▸; Zander *et al.*, 2015 ▸).

One of the major concerns in adopting the serial approach at synchrotrons was radiation damage. It has been reported that 0.3 MGy is the radiation-dose limit beyond which the information collected from a single crystal at room temperature is compromised (Nave & Garman, 2005 ▸). At XFELs, the pulse duration (femtoseconds) is sufficiently short such that the effects of radiation damage can be mitigated. An XFEL femtosecond pulse leads to photo-ionization of the inner core shell electrons, producing ‘hollow atoms’, in less than 10 fs (Hau-Riege & Bennion, 2015 ▸). Molecules are then destroyed by Coulomb explosion, which terminates diffraction before secondary radiation damage can take place. For this reason, even though XFELs produce extremely brilliant X-rays, SFX data show sufficiently reduced radiation damage to allow the collection of data at doses in excess of 3 GGy (Lomb *et al.*, 2011 ▸), which is over 10 000 times the estimated tolerable dose at room temperature (Nave & Garman, 2005 ▸). At synchrotron sources, the low monochromatic photon flux along with the quasi-continuous beam-operation mode lead to an exposure timescale per crystal/image in the millisecond range, which is not fast enough to outrun primary damage. Here, secondary radiation damage plays a major role as free radicals reduce molecules, leading to the breakage of chemical bonds, which finally destroys the molecules and weakens crystal contacts on much longer timescales than the Coulomb explosion observed with XFEL femtosecond pulses. However, a recent study has demonstrated that for the same total dose, secondary radiation damage owing to diffusion of free radicals can be reduced by increasing the intensity and inversely decreasing the exposure time (Owen *et al.*, 2012 ▸). Another factor that has contributed to the success of SMX at synchrotrons is the recent improvement in sample-delivery methods (for example the high-viscosity injector), and the high efficiency achieved with fast-readout detectors that can collect data in a shutterless mode. High-viscosity injectors require reduced sample volumes compared with liquid injectors (for example the gas dynamic virtual nozzle; GDVN; Weierstall *et al.*, 2014 ▸). In addition, fast-flowing liquid injectors are impractical for SMX experiments because the crystal transit time across the beam would be in the submillisecond range and thus the crystal would not be exposed to sufficient flux to diffract strongly. A large variety of sample-delivery methods have been successfully applied, including delivering crystals by flowing them through capillaries (Stellato *et al.*, 2014 ▸), high-viscosity injectors (Botha *et al.*, 2015 ▸; Nogly *et al.*, 2015 ▸) and novel fixed-target systems (Coquelle *et al.*, 2015 ▸; Gati *et al.*, 2014 ▸; Hasegawa *et al.*, 2016 ▸; Huang *et al.*, 2015 ▸; Murray *et al.*, 2015 ▸).

With the intention of making the serial crystallography approach more broadly accessible to the structural biology community, we implemented SMX at the GM/CA 23-ID-D beamline at the Advanced Photon Source (APS), a third-generation synchrotron source at Argonne National Laboratory, Chicago, Illinois, USA. A set of proteins was chosen including membrane proteins, soluble proteins and a multi-protein cofactor complex. The membrane protein was the human A_2A_ adenosine receptor containing the BRIL fusion protein in the third intracellular loop (A_2A_AR). A_2A_AR is one of the best-studied human G-protein coupled receptors (GPCRs) for which a structure has been determined previously (Batyuk *et al.*, 2016 ▸; Liu *et al.*, 2012 ▸). The set of soluble proteins that we tested include *Francisella tularensis* SCHUS4 Flpp3 soluble domain, proteinase K, thaumatin and lysozyme, and the multiprotein cofactor antenna complex phycocyanin (PC). PC is a cyanobacterial antenna protein that forms part of the phycobilisome light-harvesting complex, which channels excitation energy to photosystem II. The PC complex forms a disk-like trimer in which each monomer is composed of two subunits, α and β (Schirmer *et al.*, 1985 ▸), where each subunit binds three chromophores. Flpp3 is the soluble domain of a membrane lipoprotein located in the outer membrane of *F. tularensis*, which is the causative agent of the disease tularemia. Flpp3 has been identified as a virulence determinant (Su *et al.*, 2007 ▸), and its atomic resolution structure has recently been determined by NMR (Zook *et al.*, 2015 ▸); however, a crystallographic structure has not yet been reported. Proteinase K is a commercially available enzyme belonging to the family of serine proteases, which has been used in numerous studies to identify disordered regions in intrinsically disordered proteins (IDPs; Csizmók *et al.*, 2005 ▸; Denning *et al.*, 2003 ▸; Marín *et al.*, 2012 ▸; Martin-Garcia *et al.*, 2014 ▸; Nyarko *et al.*, 2004 ▸). Thaumatin and lysozyme were also included as they crystallize rapidly and easily, and are therefore frequently used as model systems in protein crystallization studies and many proof-of-principle studies.

The goal of this study was not to determine the structures of these proteins, but to evaluate the feasibility of SMX experiments by using the current capabilities of the GM/CA 23-ID-D beamline in combination with various delivery methods. For this, microrystals in a size range from 5 to 20 µm were grown and delivered to the X-ray beam using a high-viscosity injector (Weierstall *et al.*, 2014 ▸). We also tested two viscous media as crystal carriers: lipidic cubic phase (LCP) and poly(ethylene oxide) (PEO), a gel polymer with a high molecular weight (8 000 000). Additionally, we developed a new program at APS for real-time data monitoring and reduction, as well as a new version of the *Cheetah* software (*Cheetah-cbf*) for data reduction (hit-finding) that reads raw images from the PILATUS detector. In this proof-of-principle study, we determined the feasibility of structure determination of several proteins, including A_2A_AR, PC, Flpp3, proteinase K and lysozyme, using SMX. Much faster data collection will be possible using the upgraded high-brightness synchrotron sources and faster detectors that are under way or planned at many facilities.

## 2. Materials and methods   

### 2.1. Microcrystal sample preparation   

A total of six proteins were screened in this study: the human A_2A_ adenosine receptor containing the BRIL fusion protein in the third intracellular loop (A_2A_AR); PC from *Thermosynechococcus elongatus*; Flpp3 from *F. tularensis*; chicken egg-white lysozyme; thaumatin from *Thaumatococcus daniellii*; and proteinase K from *Tritirachium album*.

Protein production and crystal sample preparations of all proteins screened were as follows. Expression, purification and crystallization of human A_2A_AR were performed as described previously (Batyuk *et al.*, 2016 ▸; Liu *et al.*, 2012 ▸). Briefly, concentrated protein (25 mg ml^−1^) was reconstituted in LCP by mixing with molten monoolein/cholesterol [9:1(*w*/*w*)] using a syringe mixer (Cheng *et al.*, 1998 ▸). Crystals of approximately 5 µm were obtained in Hamilton syringes by injecting 6 µl of protein–LCP mixture into a 100 µl syringe filled with 60 µl of a precipitant solution consisting of 0.1 *M* sodium citrate pH 5.0, 32% PEG 400, 0.075 *M* sodium thiocyanate. Flpp3 was purified as described previously (Zook *et al.*, 2015 ▸). 10–15 µm crystals of Flpp3 were grown by incubating the protein at 20 mg ml^−1^ in a low ionic strength buffer (0.05 *M* NaCl, 0.02 *M* Tris pH 6.4) containing 1 U ml^−1^ factor Xa protease to cleave the His tag. Chicken egg-white lysozyme (catalog no. 62970, Sigma) crystals of sizes between 5 and 10 µm were grown using the batch and vapor-diffusion methods. For the batch method, 40 µl of lysozyme at 50 mg ml^−1^ in 0.02 *M* sodium acetate pH 4.6 was mixed with 200 µl of a precipitant solution consisting of 18%(*w*/*v*) NaCl, 6%(*w*/*v*) PEG 6000, 0.1 *M* sodium acetate pH 3.0. Lysozyme microcrystals formed immediately at room temperature and were centrifuged at 500*g* for 5 min. For the vapor-diffusion method, lysozyme was crystallized from a kit purchased from Hampton Research (catalog no. HR7-108). A protein stock solution was made at a concentration of 100 mg ml^−1^ in deionized water. Hanging drops consisted of equal volumes of either 80 or 100 mg ml^−1^ protein solution and reservoir solution consisting of 30%(*w*/*v*) PEG 5000 monomethyl ether, 1.0 *M* sodium chloride, 0.05 *M* sodium acetate trihydrate pH 4.6. Thaumatin was purchased from Sigma (catalog No. T7638) to grow microcrystals of ∼20 µm in size using the vapor-diffusion method at room temperature by mixing equal volumes of a protein solution at 40 mg ml^−1^ and a precipitant solution consisting of 0.1 *M* sodium citrate pH 5.6, 0.75 *M* potassium/sodium tartrate. Proteinase K (catalog No. P2308, Sigma) was crystallized using the batch method by mixing equal volumes of a protein solution at 40 mg ml^−1^ in 0.02 *M* MES pH 6.5 and a precipitant solution composed of 0.1 *M* MES pH 6.5, 0.5 *M* sodium nitrate, 0.1 *M* calcium chloride. Crystals between 10 and 15 µm in size appeared after 20 min incubation at 20°C. PC was isolated from *T. elongatus* cells and purified as described previously (Fromme *et al.*, 2015 ▸). PC crystals (∼20 µm) were produced at 4°C using the batch method at a starting protein concentration of 20 mg ml^−1^ and using 0.075 *M* HEPES pH 7.0, 0.02 *M* MgCl_2_, 9%(*w*/*v*) PEG 3350 as precipitant. Crystals were grown at 4°C, appeared within 12 h and were pooled together before mixing with the viscous media. Except for A_2A_AR, all crystallization setups were carried out onsite at the experimental laboratory of GM/CA beamline 23-ID-D.

### 2.2. Mixing crystals with the injection carrier media   

**Figure 1:**
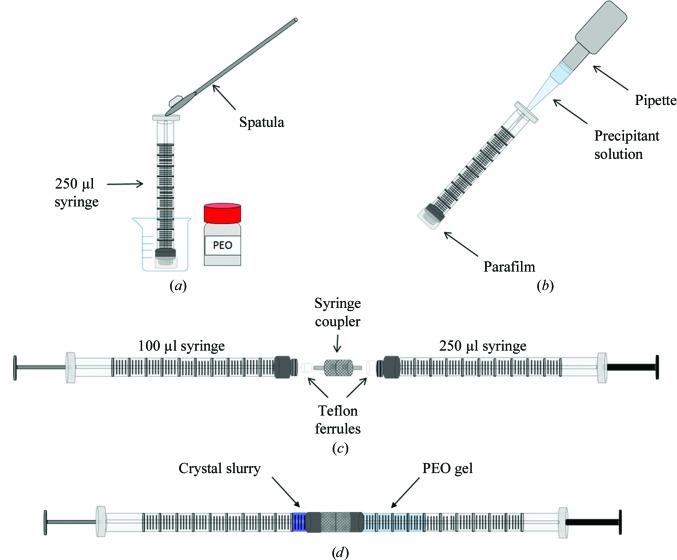
Diagram depicting the procedure used to prepare the poly(ethylene oxide) (PEO) gel and embed the crystals within it. (*a*) 0.6 mg of PEO is weighed into the back end of a 250 µl syringe and the PEO powder is then evenly distributed throughout the syringe by tapping the syringe while the syringe is horizontal (not shown). (*b*) The crystal precipitant (50 µl) is then added *via* a pipette to the back end of the syringe and the plunger is inserted. (*c*) The 250 µl syringe containing the precipitant solution and PEO is then connected to a 100 µl syringe *via* a syringe coupler (Cheng *et al.*, 1998 ▸) and the PEO is mixed until the suspension becomes clear by pushing the plungers of each syringe back and forth. The desired amount of PEO gel is then pushed into the 100 µl syringe and the syringe coupler is disconnected. (*d*) A second 100 µl syringe containing a concentrated slurry of crystals is connected to the 100 µl syringe containing the PEO gel and the crystals are then embedded into the gel by pushing the plungers back and forth until the mixture is homogenous.

A_2A_AR crystals were grown in LCP and injected directly into the X-ray beam (LCP-embedded microcrystals of A_2A_AR are shown in Supplementary Fig. S1). For the other proteins, two viscous media were used as carriers to deliver the crystals into the X-ray beam path. LCP was used to embed previously grown crystals of lysozyme (Supplementary Fig. S1), thaumatin and Flpp3. Crystal suspensions of approximately 20 µl were pooled together and centrifuged at 500*g* for 5 min. The supernatant was removed and the remaining microcrystal suspension was mixed with molten monoolein lipid in a lipid:protein ratio of 3:2(*v*:*v*) using a dual-syringe lipid mixer until a homogeneous and transparent LCP was formed (Caffrey & Cherezov, 2009 ▸; Cheng *et al.*, 1998 ▸). PEO was used for the first time as a carrier to suspend previously formed crystals of Flpp3, PC and proteinase K. Fig. 1 ▸ shows the setup with which the crystals were embedded into the PEO medium. Prior to mixing with PEO, all crystal suspensions were centrifuged at 500*g* for 5 min at 20°C. A 13%(*w*/*v*) PEO gel was prepared by inserting 6.5 mg PEO powder (Sigma–Aldrich) into the back end of a 250 µl syringe with a ferrule in place and with Parafilm covering the removable needle end. Subsequently, 50 µl of the corresponding precipitant solution was pipetted into the back end of the syringe, the plunger was inserted and the syringe was flipped upside down and moved up and down to remove air and to dissolve the PEO. To achieve a homogeneous PEO suspension, a 100 µl syringe was connected to the 250 µl syringe using a syringe coupler (Cheng *et al.*, 1998 ▸) and the plungers of both syringes were then pushed back and forth until a fully clear suspension was achieved. In another 100 µl syringe, the desired volume of crystals was drawn from the crystal pellet. Finally, a 4:1 volumetric ratio of PEO gel and concentrated crystals were mixed by pushing the crystals and gel through a syringe coupler (Cheng *et al.*, 1998 ▸) back and forth until homogeneity was achieved. Supplementary Fig. S2 shows PC and proteinase K crystals after being mixed with the PEO gel.

In all cases the crystal concentration was adjusted so that mainly single-crystal hits were observed. The crystal mixtures were loaded directly from the Hamilton syringe into the LCP injector sample reservoir.

### 2.3. Serial data collection on the GM/CA 23-ID-D beamline   

Serial data collection was performed on the GM/CA 23-ID-D beamline at the APS. The GM/CA beamline is equipped with a quad-minibeam collimator that provides high-intensity beams between 5 and 20 µm in diameter with a scatter-guard aperture (Fischetti *et al.*, 2009 ▸; Sanishvili *et al.*, 2008 ▸); the *JBluIce–EPICS* GUI software allows users to fully exploit the rapid beam-size change and various models of data collection (Hilgart *et al.*, 2011 ▸; Pothineni *et al.*, 2014 ▸; Stepanov *et al.*, 2011 ▸). A new optical layout has been designed and will be installed shortly to produce intense microfocused beams as small as 1 µm (Fischetti *et al.*, 2013 ▸).

**Figure 2:**
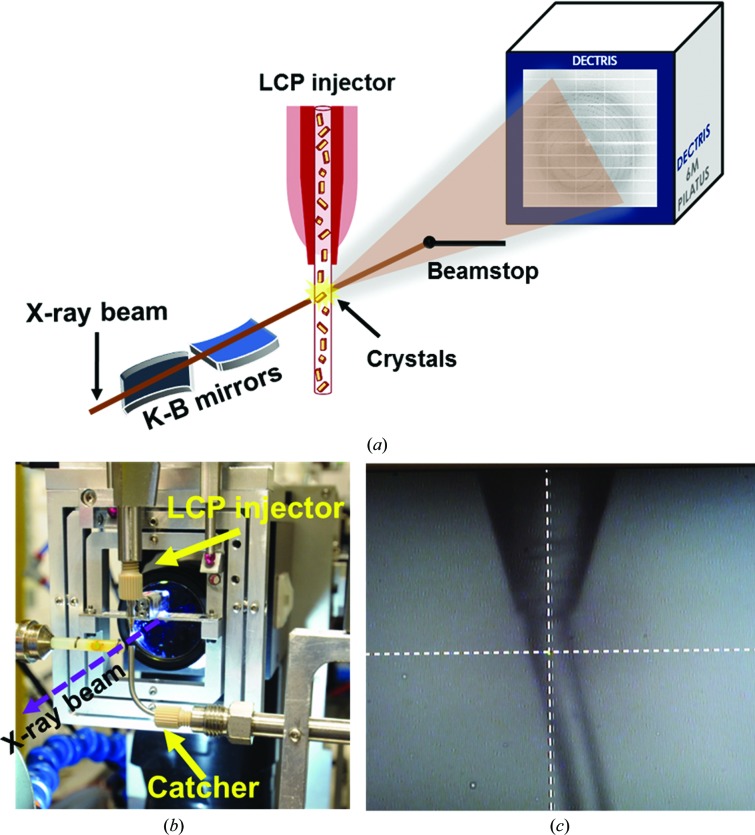
Experimental setup at the GM/CA 23-ID-D beamline. (*a*) Schematic diagram of the setup. (*b*) LCP injector (Weierstall *et al.*, 2014 ▸) mounted on translation stages (not shown). The catcher is also shown. (*c*) View of the LCP stream extruding out of a 50 µm glass capillary nozzle. The intersection point of the two white dashed lines indicates the position of the X-ray beam.

Microcrystal/carrier suspensions were injected to the intersection with the X-ray beam using the viscous LCP injector (Weierstall *et al.*, 2014 ▸) with a 50 µm inner diameter fused silica capillary (‘nozzle’). This type of nozzle was chosen according to the crystal size to avoid shearing and breakage of the crystals during injection and to avoid nozzle clogging, as well as to minimize the background scattering from crystal carrier streams. For all our experiments a 20 µl reservoir was used. The injector was mounted on translation stages to align the jet perpendicular to the X-ray beam path (Fig. 2 ▸). The crystal carrier stream was extruded out of the nozzle by a pressure that varied between 0.2 and 1 MPa depending on the flow rate of the sample, which was also varied depending on the sample composition and the observed diffraction. A nitrogen-gas sheath was introduced at the point of extrusion to prevent curling of the viscous medium stream. An in-line high-resolution microscope was used to align the nozzle to the beam and to observe the stream. A sample catcher consisting of a small diameter hyperdermic tube was placed opposite to the jet to catch the extruded waste sample. The catcher was connected to a vacuum pump *via* Tygon tubing with an inline particulate trap to protect the pump. The small tube tended to clog and was replaced by a ¼′′ tube. A schematic of the experimental setup used at the GM/CA 23-ID-D beamline is shown in Fig. 2 ▸.

During our two experiments, the beam generated by the undulator was monochromated to 0.02% bandwidth using a Si(111) monochromator and focused using Kirkpatrick–Baez (K-B) mirrors. The beamline operated at an energy of 12 keV (wavelength 1.03 Å) and the flux at the focus using a 10 µm (FWHM) beam size was 3.0–4.1 × 10^11^ photons s^−1^. Tens to hundreds of thousands of single-shot diffraction patterns of randomly oriented microcrystals were recorded on a PILATUS3 6M detector running in a continuous shutterless mode at a readout rate of 10 Hz (100 ms exposure time) while crystals were passing through the beam. The sample-to-detector distance varied between 300 and 550 mm depending on the sample diffraction quality. Although the crystal flow rate was varied based on the observed diffraction, most measurements were performed at an average flow rate which varied from 17 to 182 nl min^−1^ depending on the crystal density, corresponding to an average jet velocity of between 120 and 1250 µm s^−1^ in the 50 µm diameter nozzle. Thus, we estimated that the exposure time of a single microcrystal across the 10 µm X-ray beam was between 8 ms (for a flow rate of 182 nl min^−1^) and 85 ms (for a flow rate of 17 nl min^−1^).

### 2.4. Data processing, model building and refinement   

Data analysis is one of the major challenges of the serial crystallography approach owing to the serial nature of data collection from randomly oriented crystals of varying size and quality, the snapshot diffraction patterns of which consist of reflections measured with unknown partiality. In addition, the data collected during an experiment consist of actual crystal diffraction patterns and empty patterns (no crystals in the beam), resulting in the generation of terabytes of data. In order to monitor the hit rate and quality of large amounts of data in real time, we developed a real-time Python-based hit finder at APS. This program uses multiple processors to perform background subtraction and basic spot finding on each image as soon as it is transferred from the detector. Data reduction was also performed offsite using a modified version of *Cheetah* (Barty *et al.*, 2014 ▸), called *Cheetah-cbf*, designed to read raw cbf images from the PILATUS detector and identify crystal diffraction patterns (hits) in HDF5 format, the suitable format for data processing with *CrystFEL*. *Cheetah-cbf* was installed on the GM/CA 23-ID-D beamline; it is accessible to all APS users and can also be used offsite. For each sample the peak-finding thresholds were carefully tailored to maximize the number of crystal hits and the number of Bragg peaks in each diffraction pattern.

Sorted diffraction patterns identified during the hit-finding process were subjected to indexing and structure-factor integration using *CrystFEL* v.0.6.2 (White *et al.*, 2012 ▸, 2013 ▸, 2016 ▸). *CrystFEL* contains widely used algorithms such as *MOSFLM* (Powell *et al.*, 2013 ▸), *DirAx* (Duisenberg, 1992 ▸) and *XDS* (Kabsch, 2010 ▸), and a new algorithm known as *asdf*, which was recently implemented in *CrystFEL*. The new indexing option called ‘retry’, which was recently implemented in *CrystFEL* (White *et al.*, 2016 ▸), allowed a significant increase in the number of successfully indexed patterns. This new option rejects a small fraction of the weakest spots and retries indexing. After each pattern was indexed, the intensities were merged and integrated using a Monte Carlo algorithm (Kirian *et al.*, 2010 ▸, Kirian *et al.*, 2011 ▸). Data-collection statistics for all of the proteins tested in this study are summarized in Table 1 ▸. The resolution-cutoff criteria were based on signal-to-noise ratios, completeness and correlation coefficients. The maximum radiation dose per crystal was estimated using the *RADDOSE*-3*D* server (Zeldin, Brockhauser *et al.*, 2013 ▸; Zeldin, Gerstel *et al.*, 2013 ▸), assuming cuboid crystals for all four crystal dimensions tested in this study (5 × 5 × 5, 10 × 10 × 10, 15 × 15 × 15 and 20 × 20 × 20 µm), a 10 µm beamsize, a photon flux of 3.0–4.1 × 10^11^ photons s^−1^, an energy of 12 keV and exposure times of 6.5 and 85 ms, as described above. The results of this analysis are summarized in Table 1 ▸.

After integration with *CrystFEL*, the initial phases were obtained by molecular replacement with *MOLREP* (Vagin & Teplyakov, 2010 ▸) using known structures of the proteins from the PDB [the PDB entries used were 5k2c for A_2A_AR (Batyuk *et al.*, 2016 ▸), 4ziz for PC (Fromme *et al.*, 2015 ▸), 4zix for lysozyme (Fromme *et al.*, 2015 ▸), 5avj for proteinase K (Yazawa *et al.*, 2016 ▸) and 2mu4 for Flpp3 (Zook *et al.*, 2015 ▸)]. Additional remodeling performed by the *MR_protocol* algorithm in *Rosetta* (Terwilliger *et al.*, 2012 ▸) was required to obtain a sufficient phasing solution for Flpp3: loops from the published NMR structure (PDB entry 2mu4) were removed and then remodeled, with a total of 1000 new models, using fragments generated from the *Rosetta* webserver and initial phases provided by *Phaser* (McCoy *et al.*, 2007 ▸). The ten lowest energy models were then resubmitted to *Phaser*, with several sufficient solutions obtained. The best model had an LLG score of 56.8 and a TFZ of 7.2. A model was built using the *AutoBuilder* protocol in *PHENIX* (Terwilliger *et al.*, 2008 ▸), and the model was rephased for a final solution with an LLG of 300.7 and a TFZ of 14.7. Water molecules and ligands were removed from the reference structures for the phasing step. Structure refinement was carried out through multiple iterations of *REFMAC*5 (Murshudov *et al.*, 2011 ▸) to refine atomic coordinates and isotropic *B* factors. Manual inspection of the structures was carried out using *Coot* (Emsley & Cowtan, 2004 ▸) after each refinement step. The figures were prepared with *PyMOL *(Schrödinger). Data-refinement statistics for all structures solved in this study are summarized in Table 2 ▸. Electron-density maps were calculated with the *MAPS* tool in the *PHENIX* software suite (Adams *et al.*, 2010 ▸). Validation of all structures was carried out with the validation tools in the *PHENIX* software suite (Adams *et al.*, 2010 ▸).

## 3. Results and discussions   

Here, we demonstrate that the LCP injector-based SMX method is feasible at the GM/CA 23-ID-D beamline and can be used to determine the structures of a range of proteins of different sizes and types from crystal sizes between 5 and 20 µm. In addition, we introduce PEO gel as a novel carrier medium to deliver microcrystals into the X-ray beam, which can be utilized in serial crystallography experiments at synchrotron sources and XFELs. For our study, we used the high-viscosity injector developed at Arizona State University (Weierstall *et al.*, 2014 ▸) to deliver microcrystals into the 10 µm X-ray beam path. Diffraction data were collected in a continuous shutterless mode using a PILATUS3 6M detector operating at 10 Hz. The experimental setup at the beamline is shown in Fig. 2 ▸. To validate the proof-of-principle of the SMX approach, we chose a set of six proteins that includes one membrane protein, four soluble proteins and one multiprotein cofactor complex of different sizes as model systems: A_2A_AR, Flpp3, proteinase K, lysozyme, thaumatin and PC. Data from thaumatin microcrystals embedded in LCP were collected with a hit rate of 6%. However, the number of indexed patterns was insufficient to yield a complete data set for this protein. Complete data sets were obtained from microcrystals of two proteins embedded in LCP (A_2A_AR and lysozyme) and three proteins in PEO gel (PC, Flpp3 and proteinase K), which are described in further detail below.

### 3.1. Protein structures in LCP viscous medium   

#### 3.1.1. Lysozyme   

**Figure 3:**
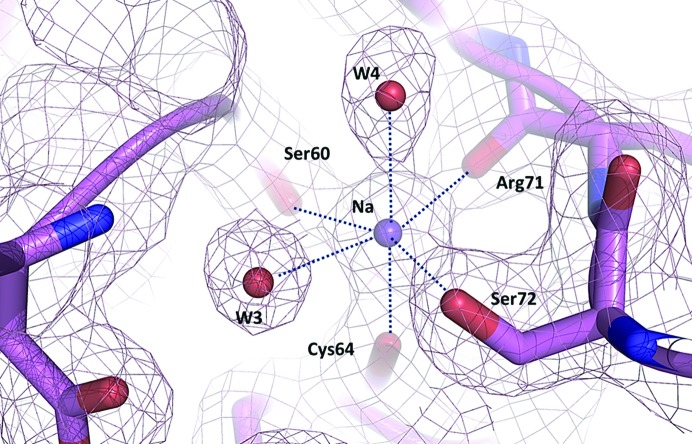
Quality of the electron-density maps displayed around the octahedral coordination of sodium in the lysozyme structure. 2*mF*_o_ − *DF*_c_ electron-density maps are shown as light pink mesh contoured at 1σ. The residues coordinating the Na atom (Ser60, Cys64, Arg71 and Ser72) are shown as a magenta stick representation. Waters W3 and W4 are shown as red spheres. The interactions between sodium and its ligands are represented as blue dotted lines.

Lysozyme was chosen as a test protein to evaluate the SMX method. Lysozyme was first crystallized and pelleted and was then reconstituted into LCP as described previously (Fromme *et al.*, 2015 ▸; Liu *et al.*, 2014 ▸). The resulting microcrystal–LCP suspension, containing lysozyme crystals of between 5 and 10 µm in size, was then transferred to the LCP injector and diffraction data were collected using an LCP flow rate of 17 nl min^−1^. Lysozyme crystals were crystallized in space group *P*4_3_2_1_2 and were occasionally seen to diffract to beyond 2.0 Å resolution (Supplementary Fig. S3). A total of 364 724 images were collected, of which 124 800 were classified as hits (hit rate 34.2%) and 18 648 were successfully indexed. The final data-collection statistics are given in Table 1 ▸. The structure of lysozyme was solved by molecular replacement with PDB entry 4zix (Fromme *et al.*, 2015 ▸) as a search model without waters or ions. The structure was refined at a resolution of 2.05 Å with an *R*_work_ and *R*_free_ of 22.8 and 26.8%, respectively (Table 1 ▸). The final refinement statistics are given in Table 2 ▸. The resulting experimental maps were of excellent quality, revealing the presence of 18 water molecules, three chloride ions and a sodium ion. The sodium ion shows an octahedral coordination by four neighboring residues and two water molecules (Fig. 3 ▸).

To ensure the validity of our procedure, the structure determined in our study was compared with those obtained by the serial method at XFELs using an LCP injector (Fromme *et al.*, 2015 ▸) and a GDVN injector (Boutet *et al.*, 2012 ▸), and at synchrotron sources using a high-viscosity injector (Botha *et al.*, 2015 ▸), glass capillaries (Stellato *et al.*, 2014 ▸) and cryocooled crystals on a fixed target (Murray *et al.*, 2015 ▸). All structures superimposed very well, with root-mean-square deviation (r.m.s.d.) values of ∼0.4 Å for C^α^ atoms (∼0.8 Å for all atoms of the protein). Only small differences were detected at the side chains of highly solvent-exposed residues, which can be explained in terms of the variation in crystal-preparation protocols and crystal-delivery methods. Analysis of the *B*-factor distribution revealed no significant differences overall between our structure (35 Å^2^) and the cryocooled structure (31 Å^2^; Murray *et al.*, 2015 ▸) and those collected at XFELs using the GDVN injector (35 Å^2^; Boutet *et al.*, 2012 ▸) and the LCP injector (27 Å^2^; Fromme *et al.*, 2015 ▸). In addition, lower *B* factors are observed for our structure compared with those determined using glass capillaries (52 Å^2^; Stellato *et al.*, 2014 ▸) and the high-viscosity injector of Botha and coworkers (48 Å^2^; Botha *et al.*, 2015 ▸) .

**Figure 4:**
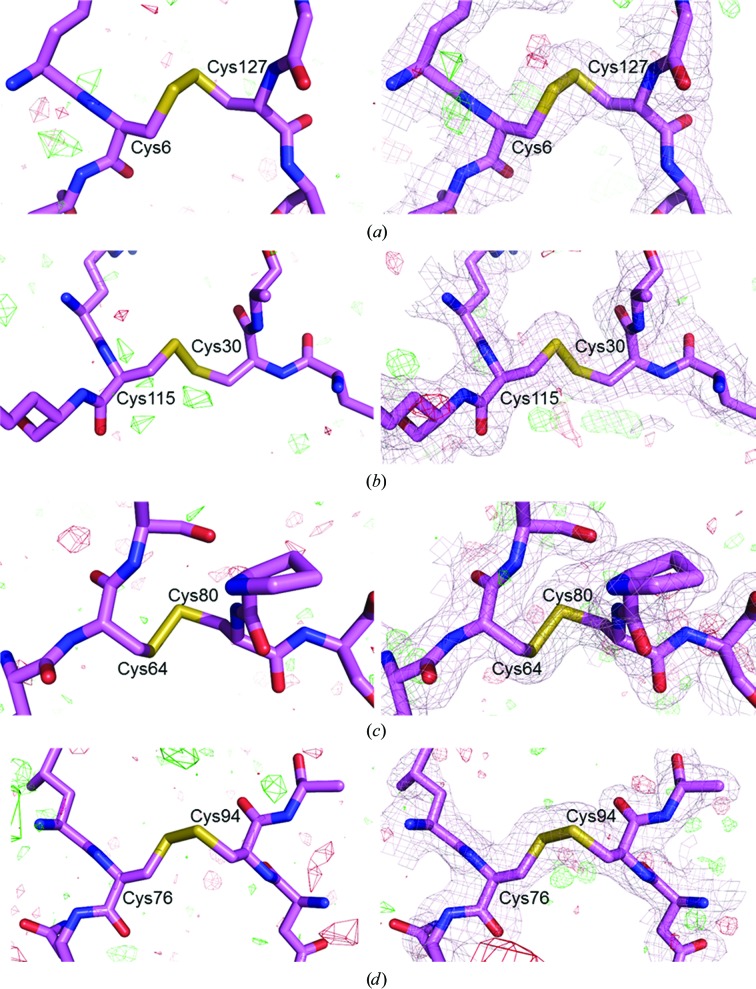
Electron-density maps displayed around the four disulfide bridges in lysozyme: (*a*) Cys6–Cys127, (*b*) Cys30–Cys115, (*c*) Cys64–Cys80 and (*d*) Cys76–Cys94. Cysteines and neighboring residues are represented as pink sticks. Left panels, structure-factor amplitude Fourier difference (*F*_o_ − *F*_o_) maps at 3σ between our data and the data set collected at LCLS (Fromme *et al.*, 2015 ▸), with red and green contours indicating negative and positive density, respectively. Right panels, 2*mF*_o_ − *DF*_c_ (light pink mesh, contoured at 1σ) and *mF*_o_ − *DF*_c_ (red and green meshes, contoured at 3σ) maps.

It has been suggested that at room temperature the radiation-dose limit above which radiation damage becomes significant is 0.3 MGy (Nave & Garman, 2005 ▸). In a recent study carried out by Coquelle and coworkers in which a room-temperature raster-scanning serial crystallography method was used at the ESRF synchrotron, the maximum dose per crystal was estimated to be between 3.2 and 29.1 MGy (Coquelle *et al.*, 2015 ▸). They demonstrated that mainly the S atoms in lysozyme and, in particular, disulfide bonds were damaged by radiation to some extent, although other structural information was not compromised (Coquelle *et al.*, 2015 ▸). In our study the average maximum radiation dose per crystal was estimated to be 0.1 MGy (Table 1 ▸), which is below the dose beyond which radiation damage is expected. In order to assess whether the disulfide bonds of our lysozyme structure were radiation-damaged considering the above radiation dose, we calculated the structure-factor amplitude Fourier difference (*F*_o_ − *F*_o_) maps between our data set and the data set collected using the LCP injector at the LCLS (Fromme *et al.*, 2015 ▸). The maximum radiation dose per crystal for the SFX data was 2.5 MGy, which is much higher than the maximum dose limit of 0.3 MGy (Nave & Garman, 2005 ▸). As shown in Fig. 4 ▸, none of the four disulfide bonds in lysozyme, including the disulfide bridge Cys64–Cys80, which was considered to be the most radiation-sensitive at room temperature by Coquelle and coworkers, show negative peaks at the S atoms or at the disulfide bridges. In addition, radiation damage was not visible in the 2*mF*_o_ − *F*_o_ and *mF*_o_ − *F*_o_ maps (Fig. 4 ▸), indicating that the disulfide bonds were not broken and the structural information has not been compromised.

#### 3.1.2. A_2A_AR   

**Figure 5:**
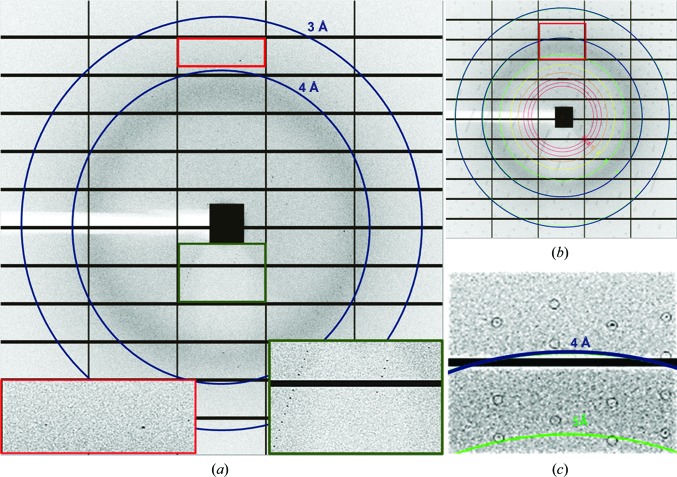
Diffraction pattern of a single microcrystal of A_2A_AR in LCP. (*a*) Raw diffraction pattern with resolution rings at 3 Å and 4 Å showing visible Bragg spots extending to about 3.5 Å resolution (red inset panel). The green inset panel shows low-resolution Bragg spots. (*b*) The same diffraction pattern as in (*a*) after indexing with resolution rings. (*c*) Closer view of the red boxed area highlighted in (*b*).

**Figure 6:**
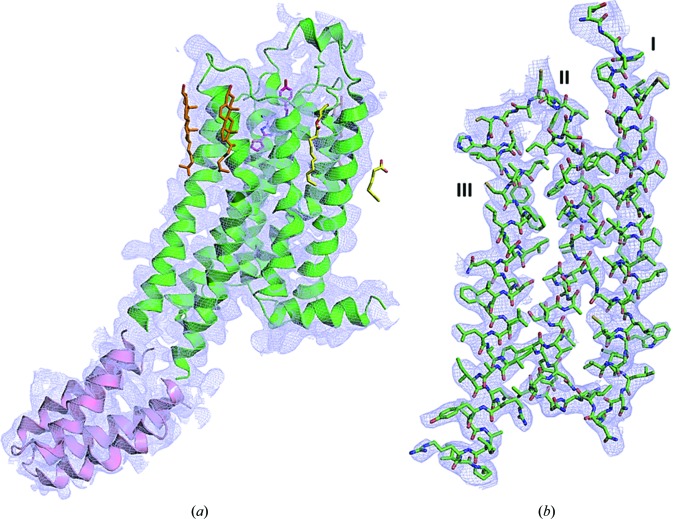
The quality of the 2*mF*_o_ − *DF*_c_ electron-density maps reflects the good quality of the collected data. (*a*) The A_2A_AR protein is shown as a green cartoon and the BRIL fusion protein is shown as a pink cartoon. The ligand ZM241385 (magenta), three cholesterols (orange) and three lipids (yellow and cyan) are shown as stick representations. (*b*) 2*mF*_o_ − *DF*_c_ electron-density maps around the residues, shown as sticks, for helices I, II and II.

Microcrystals of A_2A_AR were injected into the X-ray beam path at an average flow rate of 56 nl min^−1^ and measured for ∼14 h. Less than 55 µl of LCP–sample was necessary to collect a total of 500 003 images, of which 14 711 were identified as hits (3% hit rate) and 5287 (indexing yield 36%) were successfully indexed in space group *C*222_1_ (Table 1 ▸). Fig. 5 ▸ shows a diffraction pattern of a single A_2A_AR microcrystal. The structure of A_2A_AR was solved by molecular replacement using the recently reported high-resolution SFX structure (PDB entry 5k2c; Batyuk *et al.*, 2016 ▸) without waters and ligands. The final structure was refined to 3.2 Å resolution with an *R*_work_ and *R*_free_ of 24.2 and 28.6%, respectively (Table 1 ▸). The final data-collection and refinement statistics are given in Tables 1 ▸ and 2 ▸. Despite the medium resolution of the structure, the high quality of the electron-density maps allowed us to model the ligand ZM241385, three cholesterol molecules and three other lipids (Fig. 6 ▸). However, the clusters of water molecules previously described in the structures determined by SFX and under cryoconditions, which were demonstrated to play a key role in maintaining the stability of the receptor and in ligand binding, were not identified in our medium-resolution A_2A_AR structure. Also, the sodium ion which has been described to play a key role in the receptor-activation mechanism (Katritch *et al.*, 2014 ▸) was not identified in our structure, possibly for the same reason.

We compared the structure of A_2A_AR obtained in our study with high-resolution structures obtained by SFX (Batyuk *et al.*, 2016 ▸) and from cryocooled crystals (Liu *et al.*, 2012 ▸). All structures aligned very closely, with r.m.s.d. values for C^α^ atoms of 0.3 and 0.4 Å for the SFX and synchrotron structures, respectively. Larger differences were found around the side chains of solvent-exposed bulky residues, the orientation of which could not be well modeled. The discrepancies were slightly larger in the BRIL fusion protein, where small differences were even observed along the backbone of this protein. The average *B* factors were also significantly higher (109 Å^2^ for the whole protein, 148 Å^2^ for the BRIL fragment and 96 Å^2^ for the A_2A_AR fragment) compared with that of the room-temperature structure solved by SFX (∼40 Å^2^ for the whole protein; Batyuk *et al.*, 2016 ▸). Also, the observed high *B* factors may explain why no water molecules are seen in our structure. High average *B* factors (90–100 Å^2^) have so far been reported in the literature for 86 structures deposited in the PDB at resolutions between 3.15 and 3.25 Å. In fact, high *B* factors should be expected when solving medium- to low-resolution crystallographic structures at room temperature.

The A_2A_AR protein has 15 Cys residues in its sequence, eight of which form four disulfide bonds (Cys71–Cys159, Cys74–Cys146, Cys77–Cys166 and Cys259–Cys262), which might be subject to radiation damage. In order to assess the effect of radiation damage, we calculated the structure-factor amplitude Fourier difference (*F*_o_ − *F*_o_) maps between our data set and the undamaged data set collected at the LCLS (Batyuk *et al.*, 2016 ▸). As shown in Supplementary Fig. S4, the absence of negative electron-density peaks around disulfide bonds indicates that the radiation-damage effect is limited, as expected by the estimated radiation dose of 0.2 MGy. The 2*mF*_o_ − *F*_o_ and *mF*_o_ − *F*_o_ electron-density maps also demonstrate that the disulfide bonds are not broken (Supplementary Fig. S4).

### 3.2. Protein structures in the novel PEO viscous medium   

#### 3.2.1. Flpp3   

A highly concentrated microcrystal pellet was mixed with either LCP or PEO gel. The LCP- and PEO-embedded microcrystals were transferred to the high-viscosity injector (Weierstall *et al.*, 2014 ▸) and presented into the beam in a free-flowing stream. Diffraction data for the Flpp3 microcrystals were collected at an average flow rate of 155 nl min^−1^, consuming less than 30 µl of protein/carrier sample, which yielded 13 383 hits, of which 3157 were indexed at 3.0 Å resolution (Table 1 ▸). The final data set was generated from reflections collected from both PEO and LCP samples. Efforts to generate a sufficient model for Flpp3 at 3.0 Å resolution are ongoing. The final data-collection and refinement statistics for the Flpp3 data set are shown in Tables 1 ▸ and 2 ▸.

#### 3.2.2. Phycocyanin (PC)   

**Figure 7:**
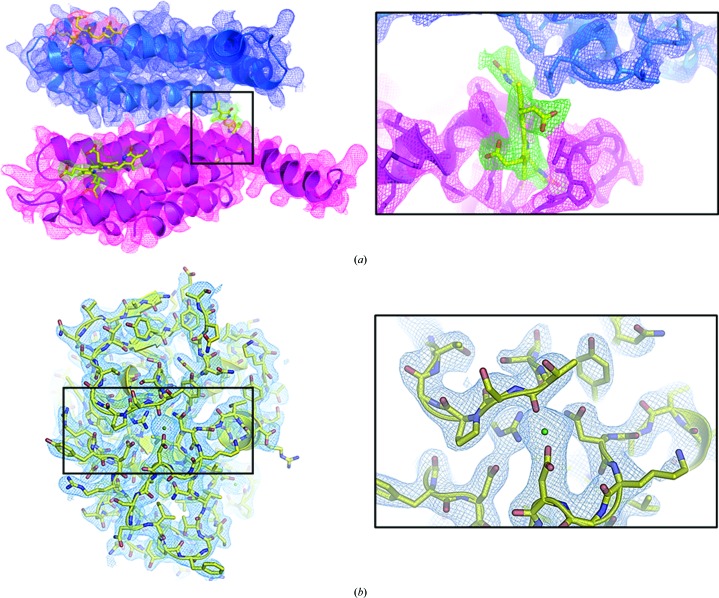
2*mF*_o_ − *DF*_c_ electron-density maps of PC (*a*) and proteinase K (*b*) contoured at 1σ. (*a*) The two PC subunits (α, blue; β, pink) are shown in cartoon representation. The three chromophores are shown as yellow sticks. A closer view of the chromophore in the black box is shown in the right panel. (*b*) Proteinase K is shown in a yellow cartoon and stick representation. One of the two Ca atoms is shown as a green sphere. A closer view of the Ca atom is illustrated in the right panel.

The PEO with embedded PC microcrystal (∼20 µm) suspension was injected at an average flow rate of 182 nl min^−1^ and 159 809 images were collected, which correspond to an effective measurement time of ∼5 h (Table 1 ▸). This resulted in a PC sample consumption of about 60 µl. The structure of PC was determined to 3.1 Å resolution from 1826 indexed patterns (indexing rate 23%; Table 1 ▸). The final data-collection and refinement statistics of the PC data set are shown in Tables 1 ▸ and 2 ▸. Supplementary Fig. S5 shows a diffraction pattern of a single PC–PEO microcrystal. Slightly smeared Bragg peaks were observed, which indicates that the PC crystals were rotating azimuthally around the X-ray beam axis during the 100 ms exposure. This has previously been observed for lysozyme microcrystals embedded in LCP with 100 ms exposures (Botha *et al.*, 2015 ▸). The structure of PC was solved by molecular replacement using the previously solved LCP-injected SFX structure as a search model, with water molecules and chromophores removed (PDB entry 4ziz; Fromme *et al.*, 2015 ▸). The PC structure was refined to 3.1 Å resolution, with final *R*_work_ and *R*_free_ values of 17.2 and 20.4%, respectively. The quality of the structure can be assessed from the 2*mF*_o_ − *DF*_c_ electron-density maps of the three chromophores of PC (Fig. 7 ▸ *a*). To further evaluate the quality of our structure, we compared our model with the two SFX structures recently described using the LCP injector (PDB entry 4ziz; Fromme *et al.*, 2015 ▸) and the GDVN injector (PDB entry 4q70; R. Fromme, S. Roy-Chowdhury, S. Basu, C. Yoon, D. Brune & P. Fromme, unpublished work). The three structures aligned very closely with each other, with r.m.s.d. values for C^α^ atoms of 0.1 and 0.7 Å for the LCP and GDVN structures, respectively. Furthermore, we compared the PC structure with the structure previously solved at a synchrotron using a single cryocooled crystal (PDB entry 3l0f; R. Fromme, D. Brune & P. Fromme, unpublished work). Both structures superimposed very well, with an r.m.s.d. value of 0.2 Å for all C^α^ atoms. An r.m.s.d. value of 0.5 Å for all atoms indicates that small differences were, however, observed in the loop regions and in the solvent-exposed regions. The average *B* factors for both chains of PC (45 Å^2^ for chain *A* and 51 Å^2^ for chain *B*) showed no significant differences from the LCP structure (38 Å^2^ for chain *A* and 45 Å^2^ for chain *B*). However, higher average *B* factors were observed compared with those of the cryocooled (21 Å^2^ for chain *A* and 25 Å^2^ for chain *B*) and the GDVN (26 Å^2^ for chain *A* and 32 Å^2^ for chain *B*) structures. These differences are attributable, to a greater extent, to the higher resolution of the structures collected at cryogenic temperatures (1.35 Å^2^; R. Fromme, D. Brune & P. Fromme, unpublished work) and using a liquid jet (1.9 Å^2^; R. Fromme, S. Roy-Chowdhury, S. Basu, C. Yoon, D. Brune & P. Fromme, unpublished work).

#### 3.2.3. Proteinase K   

Diffraction data for proteinase K (PK) microcrystals embedded in PEO gel were collected at a constant flow rate of 79 nl min^−1^ with an effective measurement time of ∼3 h, during which nearly 100 000 images were recorded. This resulted in a PK sample consumption of less than 15 µl. A total of 4497 images with diffraction to a maximum resolution of 2.2 Å were identified as hits (hit rate 4.2%), of which 817 were successfully indexed in space group *P*4_3_2_1_2 (Table 1 ▸). The final data-collection and refinement statistics are given in Tables 1 ▸ and 2 ▸. Supplementary Fig. S6 shows a diffraction pattern from a single proteinase K–PEO microcrystal. As with PC, slightly smeared Bragg peaks were also observed, which indicates that the proteinase K crystals were rotating during the 100 ms exposure. Phasing was performed by molecular replacement using the cryocooled structure at a synchrotron (PDB entry 5avj; Yazawa *et al.*, 2016 ▸) as a search model. The final refinement gave an *R*_work_ and *R*_free_ of 22.5 and 24.3%, respectively. The proteinase K structure was determined to 2.65 Å resolution and, overall, our structure is very similar to that solved using cryocooled crystals, with an r.m.s.d. value of less than 0.1 Å for all C^α^ atoms. The average *B* factor was 61 Å^2^, as expected for a structure solved to 2.65 Å resolution at room temperature. In our structure, no water molecules were identified; however, the two Ca atoms that contribute to protein stability were clearly visible. The 2*mF*_o_ − *DF*_c_ electron-density maps around the one of these two Ca atoms can be used to assess the high quality of our proteinase K structure (Fig. 7 ▸ *b*).

### 3.3. PEO gel: a novel high-viscosity medium as a crystal carrier for serial crystallography   

One of the greatest limitations of SMX experiments at synchrotron sources using monochromatic X-rays is that the crystals have to be exposed for longer times, which decreases the data-acquisition rates at these facilities (typically <20 Hz) compared with those at XFELs (120 Hz at LCLS and 60 Hz at SACLA). Delivering crystals in a liquid jet using the GDVN injector has been successfully used for many SFX experiments at XFELs (for a review, see Martin-Garcia *et al.*, 2016 ▸). The GDVN injector operates at a minimal flow rate of 10 µl min^−1^, which is too fast even for the repetition rates of current XFELs; thus, most of the sample is wasted and large sample volumes are needed to obtain a complete data set. Sample waste is even much more noticeable at the low repetition rates at synchrotrons, which makes liquid injectors impractical. Additionally, with the high flow rate of the GDVN the crystal transit of the X-ray beam is submillisecond, resulting in very weak diffraction; therefore, the GDVN is not recommended for SMX experiments. New strategies have allowed crystals to be delivered into the X-ray beam path more efficiently so that sample waste is markedly reduced. One of these methods is the use of a high-viscosity medium extruded as a continuous stream using the LCP injector (Weierstall *et al.*, 2014 ▸) at very slow flow rates (typically 1–300 nl min^−1^), allowing extremely low sample consumption (less than 1 mg) compared with a GDVN injector (10–100 mg protein).

In our study, we have used LCP as a crystal carrier medium which can also be used as a host matrix to crystallize membrane proteins (Caffrey & Cherezov, 2009 ▸). To date, the use of LCP has led to the structure determination of a wide range of membrane proteins, including ion channels, transporters, enzymes, β-barrels and, in particular, GPCRs (Xiang *et al.*, 2016 ▸). More recently, LCP has been successfully used to deliver previously grown soluble protein crystals into the X-ray beam in serial crystallography experiments, allowing their structure determination using XFELs (Fromme *et al.*, 2015 ▸) and synchrotrons (Botha *et al.*, 2015 ▸; Nogly *et al.*, 2015 ▸; Stellato *et al.*, 2014 ▸). In the case of membrane proteins, crystals have to be grown in LCP since mixing membrane-protein crystals pregrown in solution in the form of protein–detergent micelles with LCP typically leads to dissolution of the crystals. This is probably owing to partitioning of detergent molecules into the lipid bilayer of LCP, leading to crystal dissolution and protein denaturation.

**Figure 8:**
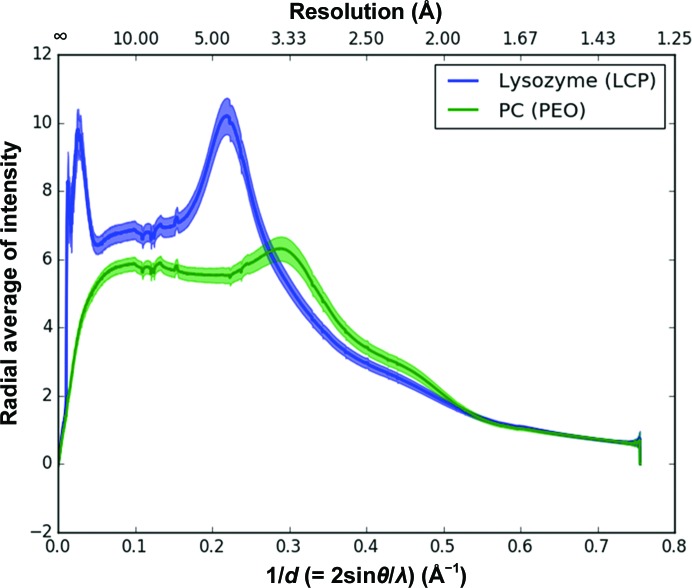
Diffuse background scattering comparison between PEO gel and LCP. The mean radial intensity over the total number of frames used for each medium protein is plotted against resolution (1/*d*). The cyan line represents the mean radial intensity for lysozyme in LCP medium as a function of 1/*d* (or resolution in Å on the upper *x* axis). The green line represents the mean radial intensity for PC in PEO gel medium as a function of 1/*d*. The error or fluctuation in the radial intensity is quantified using the mean absolute deviation of both media, which is shown as transparent regions. The lines end at different resolution points owing to the different crystal-to-detector distances.

A novel high-viscosity medium has now been presented here to deliver protein microcrystals for SMX experiments. A gel polymer of high-molecular-weight poly(ethylene oxide) (PEO) has been used as a host matrix to embed previously grown protein microcrystals of Flpp3, proteinase K and PC. PEO has the same chemical composition as PEG, a commonly used precipitating agent in protein crystallography, but owing to its high molecular weight it is referred to by a different name. PEO is one of the most studied water-soluble synthetic polymers (Bailey, 1976 ▸; Hammouda *et al.*, 2004 ▸) and is used as a model for biomedical applications such as in drug-delivery systems (Dhawan *et al.*, 2005 ▸). PEO is soluble in water over a wide range of degrees of polymerization and at moderate temperatures (20–30°C; Blank, 1974 ▸). The use of PEO as a gel has been reported for the crystallization of a wide variety of compounds from inorganic solids (Bianconi *et al.*, 1991 ▸) to organic molecules (Chandrasekhar, 2000 ▸; Choquesillo-Lazarte & García-Ruiz, 2011 ▸) and, recently, proteins (Pietras *et al.*, 2010 ▸). In contrast to Mebiol, a medium that is viscous only at temperatures above 25°C and that has recently been used as a crystal carrier (Botha *et al.*, 2015 ▸), PEO gels are highly stable at a wide range of temperatures, so that PEO-mediated crystal delivery can be accomplished at the traditional crystallization temperatures of 4–30°C. Additionally, making PEO gel is a very simple and straightforward procedure, as described in §[Sec sec2]2, leading to a highly homogeneous crystal distribution. PEO gels, like any other PEG, are compatible with a wide variety of precipitants commonly used in protein crystallization and have also been shown to be compatible with a wide variety of organic solvents that are commonly used as additives in many crystallization recipes (Choquesillo-Lazarte & García-Ruiz, 2011 ▸). In all of our experiments, PEO-embedded crystals established very stable streams and no signs of dehydration were observed. Fig. 8 ▸ shows a comparison of the diffuse background X-ray scattering of the PEO gel stream compared with an LCP stream under the same experimental conditions. Details of the analysis used to evaluate background scattering is included in the Supporting Information. The characteristic broad peak at about 4.5 Å resolution corresponding to diffuse scattering of the LCP stream can be seen in Fig. 8 ▸. Diffuse scattering from PEO gel is observed in the 3.3 Å resolution region; it is mostly dominated by water scattering owing to the low PEO content. Overall, the background scattering from PEO gel medium is roughly 1.5 times less than that from LCP in the diffuse-ring regions (resolutions between 3.5 and 6 Å). In addition, unlike PEO gel, LCP shows strong scattering at resolutions of less than 30 Å, which makes PEO gel an ideal crystal carrier for protein crystals with large unit cells. A similar diffuse scattering profile to that of PEO gel has previously been reported for agarose medium (Conrad *et al.*, 2015 ▸). This is not surprising considering that both PEO and agarose gels have a high water content (over 90%) compared with that of LCP (∼50%). Therefore, agarose and PEO have been demonstrated to be highly stable viscous media, and are compatible with a wide variety of crystallization compounds, making them suitable as crystal carriers.

Preliminary studies indicate that PEO gel is also suitable for mixing with crystals of membrane proteins such as photosystem I (PSI), a large membrane-protein complex of 1080 kDa containing 36 protein subunits and more than 300 cofactors. Observation of PSI crystals immersed in PEO by ultraviolet two-photon excited fluorescence (UV-TPEF), polarized light and SONICC (Kissick *et al.*, 2011 ▸) imaging indicate that PSI crystals remain intact after mixing with PEO (Supplementary Fig. S7).

## 4. Conclusions and outlook   

Here, we present a proof-of-concept demonstration for the data collection of X-ray diffraction data sets suitable for structure determination from macromolecular, micrometre-sized crystals at room temperature using a high-viscosity injector on the GM/CA 23-ID-D beamline at APS. The number of crystallographic experiments carried out at room temperature has repaidly grown since the first SFX experiment was conducted at an XFEL in 2009 (Chapman *et al.*, 2011 ▸). XFELs offer unique X-ray beam properties (ultrashort, extremely intense pulses with a high repetition rate and coherence), which enables the determination of room-temperature structures, outrunning most radiation damage. However, the number of XFEL sources is limited, and therefore the structural biology community has begun to adapt the serial crystallography method at conventional synchrotron sources. Even though synchrotron sources are not as powerful as XFELs, they can produce sufficiently bright X-ray beams to enable data to be collected from 5–20 µm-sized crystals on millisecond timescales. Also, recent and future developments and upgrades in optics, sample delivery, detector technology and synchrotrons themselves will make these radiation sources even more suitable for serial data collection. Synchrotron sources themselves are more accessible worldwide compared with XFELs. For all of these reasons, the number of SMX experiments is increasing rapidly (Botha *et al.*, 2015 ▸; Coquelle *et al.*, 2015 ▸; Gati *et al.*, 2014 ▸; Hasegawa *et al.*, 2017 ▸; Heymann *et al.*, 2014 ▸; Huang *et al.*, 2015 ▸, 2016 ▸; Murray *et al.*, 2015 ▸; Nogly *et al.*, 2015 ▸; Stellato *et al.*, 2014 ▸; Zander *et al.*, 2015 ▸).

The work presented here is an important step towards adapting the serial approach for room-temperature synchrotron data collection from a broad range of protein targets. The use of high-viscosity crystal carrier media coupled with a fast-framing detector allowed us not only to synchronize a moving crystal through an intersecting X-ray beam with the desired exposure time, but also to considerably reduce sample consumption by a factor of 20 compared with SFX experiments with the GDVN injector. The utility of a high-viscosity stream for delivering crystals in the SMX approach has been demonstrated previously, including the structure determination of bacteriorhodopsin (bR) at the ESRF (Nogly *et al.*, 2015 ▸) and the structure determination of lysozyme at SLS (Botha *et al.*, 2015 ▸). High-viscosity injectors such as that used in this study (Weierstall *et al.*, 2014 ▸) offer the possibility of using different viscous materials other than LCP as a crystal carrier medium. Embedding pregrown crystals in a high-viscosity medium may alter the properties of the matrix or even dissolve the crystals. Thus, the choice of the crystal carrier can be critical to the success of serial crystallography experiments. In our study, we have explored LCP medium as a crystal carrier medium and have shown that PEO gel is also suitable as a general delivery system for soluble proteins for experiments at synchrotrons as well as XFELs. Based on the visual techniques mentioned, PEO gels might also be suitable to deliver membrane-protein crystals in serial crystallography experiments.

Owing to the low flux density currently achievable at synchrotron beamlines compared with that of XFELs, we decided to use crystal sizes that varied from 5 to 20 µm, which allowed us to collect a complete SMX data set for four proteins by using no more than 60 µl of protein–carrier sample. The current implementation of the serial synchrotron strategy will improve with the future upgrades at the APS, which will create a smaller, brighter microfocused beam with an intensity that is up to two orders of magnitude higher. These improvements, along with new developments in beamline optics and the acquisition of faster frame-readout detectors (*e.g.* EIGER), will allow X-ray structure determination from much smaller crystals, possibly on the submicrometre scale. These improvements will probably improve the resolution of, for example, crystals of A_2A_AR using a similar crystal size (∼5 µm) from the 3.2 Å resolution obtained in this study to possibly better than 2.5 Å resolution. Another interesting prospect for serial crystallography at synchrotron sources is to use a broad-bandpass beam such as that at the BioCARS beamline at the APS. Instead of using a monochromator, a mirror is used as a low-pass filter, creating a ‘pink beam’, which can be 500-fold more intense than a monochromated beam. Preliminary experiments at BioCARS have shown that serial crystallography data can be collected with a single 100 ps pulse when the APS is operating in hybrid mode or with four bunches (460 ns) in standard 24-bunch mode (Martin-Garcia *et al.*, in preparation). However, larger crystals were required than for this monochromatic study owing to the larger beam size at BioCARS compared with GM/CA. The APS-U will allow the pink beam to be focused more tightly, enabling serial crystallography on microcrystals. The wider bandwidth of the pink beam compared with a monochromatic beam enables the measurement of full reflections, which will significantly diminish the effect of partiality on the data (White *et al.*, 2013 ▸) and lead to fewer required patterns for a complete data set.

The study presented here not only demonstrates the current feasibility of the serial crystallography method for determining static structures, but also suggests its extension to time-resolved pump–probe measurements to study light-induced reactions on millisecond timescales (or faster timescales when shorter exposure times are available) from microcrystals. Traditionally, time-resolved crystallography has been performed on large single crystals mounted on a goniometer by Laue crystallography using a pink beam (for a review, see Hajdu & Johnson, 1990 ▸). Time-resolved studies at synchrotrons have always been limited by the pulse length achievable at these facilities. However, over the past twenty years, continuous improvements in this technique have allowed a reduction in the temporal resolution regime from milliseconds (Genick *et al.*, 1997 ▸) to picoseconds (Schotte *et al.*, 2012 ▸). In addition, Laue crystallography has been predominantly limited to photo-activated reactions, where, unlike diffusion-based reactions, initiation is homogeneous and rapid. However, even in photo-induced reactions the size of the crystals matter since molecules absorb the light as it diffuses through the crystal, causing a decrease in transmission with increasing path length. Thus, smaller crystals provide an advantage, allowing more synchronized reaction initiation throughout the crystal and requiring decreased pump intensities for maximal reaction initiation. Furthermore, the volume of the crystals is so small that in practice all molecules within the crystal can be illuminated by the pump. Owing to the inherent properties of XFELs, SFX has become an ideal technique to perform time-resolved experiments (TR-SFX). Initially, TR-SFX experiments were carried out using the GDVN liquid injector to deliver the crystals into the beam. More recently, Nogly and coworkers have demonstrated for the first time that time-resolved pump–probe serial crystallo­graphy is also feasible using a high-viscosity injector (Nogly *et al.*, 2015 ▸), allowing a much lower sample consumption. Therefore, the use of a high-viscosity injector in combination with a pink beam will also provide an unprecedented view into the relations between protein structure, dynamics and function at synchrotron sources.

It is important to note that radiation damage is currently inevitable when using synchrotron sources. For decades, cryo-cooling has been used to mitigate the effect of radiation damage, which is the primary factor limiting the quality of the structural information that can be obtained from a protein crystal (Garman, 2010 ▸). Several radiation-damage studies carried out on macromolecular crystals have revealed that the lifetime of the crystals can be prolonged with an increase in the beam intensity and a decrease in exposure time (Owen *et al.*, 2012 ▸; Warkentin *et al.*, 2011 ▸, 2013 ▸). These studies have also shown that global radiation damage occurs on a timescale of seconds even with a 100 ms exposure. However, radiation damage can be notably reduced by outrunning the secondary and tertiary damage effects (Owen *et al.*, 2012 ▸; Warkentin *et al.*, 2011 ▸). The new technique of serial crystallography, in which each diffraction pattern is from a different crystal, reduces radiation damage even when measuring crystals at room temperature at synchrotron sources (Coquelle *et al.*, 2015 ▸; Nogly *et al.*, 2015 ▸; Stellato *et al.*, 2014 ▸). No radiation damage was reported in the structures of lysozyme or bacteriorhodopsin from data sets collected using a liquid injector (Stellato *et al.*, 2014 ▸) or an LCP injector (Nogly *et al.*, 2015 ▸), respectively. The maximum radiation doses were estimated to be 0.3 MGy (Stellato *et al.*, 2014 ▸) and 0.7 MGy (Nogly *et al.*, 2015 ▸). However, evidence of specific radiation damage was observed at the disulfide bridges of the structure of lysozyme from data sets collected using a raster-scanning method (Coquelle *et al.*, 2015 ▸). Considerably higher doses (3.2 and 29.1 MGy) than the theoretical safe dose limit of 0.3 MGy (Nave & Garman, 2005 ▸) were used. A comparison between the lysozyme structures when delivering crystals using a jet (Stellato *et al.*, 2014 ▸) and using a fixed target (Coquelle *et al.*, 2015 ▸), which were collected on the same beamline (ID-13 at the ESRF) under similar conditions, suggests that the radiation doses per crystal are higher when immobilizing crystals in a solid support and therefore they are more subject to radiation damage. In the study presented here, the LCP injector was operated at an average flow rate of 100 nl min^−1^, which provided a constant stream of fresh crystals, so that the average exposure time per crystal was estimated to be about 30 ms. The average total radiation dose that each single crystal received was estimated to be only 0.1 MGy, which is lower than the safe dose limit reported by Garman and coworkers for room-temperature measurements (Garman & McSweeney, 2007 ▸; Nave & Garman, 2005 ▸). In fact, we could not detect any sign of radiation damage on investigating the radiation-sensitive residues in the structures of either A_2A_AR or lysozyme, demonstrating that the methods described here enable data collection at room temperature with minimal radiation damage.

SMX experiments can also be performed at cryogenic temperatures to reduce radiation damage by raster scanning and oscillation of microcrystals mounted into fixed targets (Gati *et al.*, 2014 ▸; Hasegawa *et al.*, 2016 ▸; Zander *et al.*, 2015 ▸), allowing crystals to be exposed for longer. Also, since microcrystals are rotated during data collection, SMX at cryogenic temperatures allows complete data to be collected using a much lower sample consumption compared with that of the room-temperature-based method. However, SMX at room temperature offers several advantages over the cryo-based method, namely (i) room-temperature data collection eliminates the need for cryoprotection, which is often difficult and in some in cases impossible because the cryocooling process itself can adversely reduce the crystalline order or affect the protein structure (Fraser *et al.*, 2009 ▸, 2011 ▸; Keedy *et al.*, 2014 ▸), and (ii) as mentioned above, SMX at room temperature opens the door to time-resolved pump–probe experiments in the near future. By contrast, SMX using cryocooled crystals is only limited to the determination of static structures.

Finally, our study also confirms that for strongly diffracting crystals such as lysozyme, the serial crystallography approach at current synchrotron sources provides crystal structures of reasonable quality for crystals of 5 µm and larger. However, for weakly diffracting crystals with larger unit cells such as A_2A_AR or PC, the serial approach at room temperature does not yield a resolution as high as can be obtained at XFELs or from cryocooled crystals. Weakly diffracting crystals or crystals with large unit cells will strongly benefit from further upgrades of synchrotron sources and increased X-ray flux.

## Supplementary Material

PDB reference: adenosine receptor A_2A_AR, 5uvi

PDB reference: lysozyme, 5uvj

PDB reference: phycocyanin, 5uvk

PDB reference: proteinase K, 5uvl

Supporting information including the diffuse background X-ray scattering comparison and seven figures.. DOI: 10.1107/S205225251700570X/it5011sup1.pdf

## Figures and Tables

**Table 1:** SMX data-collection statistics Values in parentheses are for the highest resolution shell.

	A_2A_AR	Lysozyme	Phycocyanin	Flpp3	Proteinase K
Viscous medium	LCP	LCP	PEO	LCP/PEO	PEO
Crystal size (µm)	∼5	5–10	∼20	∼20	10–15
Sample-to-detector distance (mm)	550	300	300	300	400
Average flow rate (nl min^−1^)	56	17	182	155	79
Average crystal velocity (µm s^−1^)	570	120	1550	1315	675
Average exposure time/crystal (ms)	18.0	85.0	6.5	7.6	15.0
Maximum dose per crystal (MGy)	0.1	0.1	0.1	0.1	0.1
Data-collection time (h)	∼14	12	∼5	2.5	∼3
Protein/carrier volume (µl)	52.3	26.0	60	27.5	13.0
Wavelength (Å)	1.03	1.03	1.03	1.03	1.03
Maximum resolution observed (Å)	3.1	2.0	2.5	1.6	2.2
Resolution (Å)	45–3.20 (3.28–3.20)	35–2.05 (2.10–2.05)	50–3.10 (3.18–3.10)	36.8–3.00 (3.08–3.00)	50–2.65 (2.72–2.65)
Space group	*C*222_1_	*P*4_3_2_1_2	*H*32	*P*2_1_	*P*4_3_2_1_2
*a*, *b*, *c* (Å)	39.4, 179.5, 140.3	79.1, 79.1, 38.0	186.4, 186.4, 60.3	30.3, 52.3, 36.9	68.3, 68.3, 108.2
α, β, γ (°)	90, 90, 90	90, 90, 90	90, 90, 120	90, 92, 90	90, 90, 90
No. of collected images	503006	364724	159809	115672	97772
No. of hits/indexed patterns	14711/5287	124800/18648	7912/1826	13383/3157	4497/817
〈*I*/σ(*I*)〉	7.7 (0.3)	11.2 (0.4)	7.6 (1.9)	5.1 (2.5)	4.6 (0.3)
Multiplicity	142.6 (33.2)	873.3 (43.9)	156.4 (4.8)	45.1 (13.0)	104.8 (40.6)
Completeness (%)	99.8	99.8	99.7	100	99.2
CC* (%)	0.992 (0.423)	0.978 (0.436)	0.986 (0.799)	0.973 (0.856)	0.954 (0.431)
*R*_split_ (%)	13.4 (506.0)	10.7 (242.8)	14.2 (73.09)	25.9 (38.8)	22.4 (331.0)

**Table 2:** SMX data-refinement statistics

	A_2A_AR	Lysozyme	Phycocyanin	Proteinase K
Total reflections	25266	29412	17002	29685
No. of reflections used in refinement	7702	7164	6280	5294
*R*_work_/*R*_free_ (%)	24.8/28.6	22.8/26.8	17.2/20.5	22.5/24.3
No. of atoms	3140	1023	2626	2041
Protein	2989	1002	2499	2032
Water and others (ligands or ions)	151	21	127	9
Average *B* value (Å^2^)	109.2	34.9	48.2	61.3
R.m.s. deviations from ideal values				
Bonds (Å)	0.009	0.013	0.011	0.008
Angles (°)	1.614	1.306	2.093	1.126
Ramachandran plot statistics (%)				
Favored	98.9	97.6	98.8	97.1
Allowed	1.1	2.4	1.2	2.5
Disallowed	0	0	0	0.4
Rotamer outliers	0	1	2	0
PDB code	5uvi	5uvj	5uvk	5uvl

## References

[bb1] Adams, P. D. *et al.* (2010). *Acta Cryst.* D**66**, 213–221.

[bb2] Ayvazyan, V. N. *et al.* (2006). *Eur. Phys. J. D*, **37**, 297–303.

[bb3] Bailey, F. E. Jr (1976). *Poly(ethylene Oxide)*. New York: Academic Press.

[bb4] Barty, A., Kirian, R. A., Maia, F. R. N. C., Hantke, M., Yoon, C. H., White, T. A. & Chapman, H. (2014). *J. Appl. Cryst.* **47**, 1118–1131. 10.1107/S1600576714007626 PMC4038800 24904246

[bb5] Batyuk, A. *et al.* (2016). *Sci. Adv.* **2**, e1600292. 10.1126/sciadv.1600292 PMC5035125 27679816

[bb6] Bianconi, P. A., Lin, J. & Strzelecki, A. R. (1991). *Nature (London)*, **349**, 315–317.

[bb7] Blank, Z. R. A. C. & Reimschuessel, A. C. (1974). *J. Mater. Sci.* **9**, 1815–1822.

[bb8] Botha, S., Nass, K., Barends, T. R. M., Kabsch, W., Latz, B., Dworkowski, F., Foucar, L., Panepucci, E., Wang, M., Shoeman, R. L., Schlichting, I. & Doak, R. B. (2015). *Acta Cryst.* D**71**, 387–397. 10.1107/S1399004714026327 25664750

[bb9] Boutet, S. *et al.* (2012). *Science*, **337**, 362–364.

[bb10] Caffrey, M. & Cherezov, V. (2009). *Nat. Protoc.* **4**, 706–731. 10.1038/nprot.2009.31 PMC2732203 19390528

[bb11] Chandrasekhar, R. (2000). *J. Mater. Sci. Lett.* **19**, 1801–1803.

[bb12] Chapman, H. N. *et al.* (2011). *Nature (London)*, **470**, 73–77.

[bb13] Cheng, A., Hummel, B., Qiu, H. & Caffrey, M. (1998). *Chem. Phys. Lipids*, **95**, 11–21. 10.1016/s0009-3084(98)00060-7 9807807

[bb14] Choquesillo-Lazarte, D. & García-Ruiz, J. M. (2011). *J. Appl. Cryst.* **44**, 172–176.

[bb15] Conrad, C. E. *et al.* (2015). *IUCrJ*, **2**, 421–430. 10.1107/S2052252515009811 PMC4491314 26177184

[bb16] Coquelle, N., Brewster, A. S., Kapp, U., Shilova, A., Weinhausen, B., Burghammer, M. & Colletier, J.-P. (2015). *Acta Cryst.* D**71**, 1184–1196. 10.1107/S1399004715004514 PMC4427202 25945583

[bb17] Csizmók, V., Bokor, M., Bánki, P., Klement, É., Medzihradszky, K. F., Friedrich, P., Tompa, P. & Tompa, P. (2005). *Biochemistry*, **44**, 3955–3964. 10.1021/bi047817f 15751971

[bb18] Denning, D. P., Patel, S. S., Uversky, V., Fink, A. L. & Rexach, M. (2003). *Proc. Natl Acad. Sci. USA*, **100**, 2450–2455. 10.1073/pnas.0437902100 PMC151361 12604785

[bb19] Dhawan, S., Dhawan, K., Varma, M. & Sinha, V. R. (2005). *Pharm. Technol.* **29**, 82–95.

[bb20] Duisenberg, A. J. M. (1992). *J. Appl. Cryst.* **25**, 92–96.

[bb21] Emsley, P. & Cowtan, K. (2004). *Acta Cryst.* D**60**, 2126–2132. 10.1107/S0907444904019158 15572765

[bb22] Feld, G. K. *et al.* (2015). *J. Appl. Cryst.* **48**, 1072–1079.

[bb23] Fischetti, R. F., Xu, S., Yoder, D. W., Becker, M., Nagarajan, V., Sanishvili, R., Hilgart, M. C., Stepanov, S., Makarov, O. & Smith, J. L. (2009). *J. Synchrotron Rad.* **16**, 217–225. 10.1107/S0909049508040612 PMC2725011 19240333

[bb24] Fischetti, R. F., Yoder, D., Xu, S., Makarov, O., Ogata, C. & Smith, J. L. (2013). *J. Phys. Conf. Ser.* **425**, 012006. 10.1088/1742-6596/425/1/012006 PMC4222077 25383086

[bb25] Fraser, J. S., Clarkson, M. W., Degnan, S. C., Erion, R., Kern, D. & Alber, T. (2009). *Nature (London)*, **462**, 669–673. 10.1038/nature08615 PMC2805857 19956261

[bb26] Fraser, J. S., van den Bedem, H., Samelson, A. J., Lang, P. T., Holton, J. M., Echols, N. & Alber, T. (2011). *Proc. Natl Acad. Sci. USA*, **108**, 16247–16252. 10.1073/pnas.1111325108 PMC3182744 21918110

[bb27] Fromme, R., Ishchenko, A., Metz, M., Chowdhury, S. R., Basu, S., Boutet, S., Fromme, P., White, T. A., Barty, A., Spence, J. C. H., Weierstall, U., Liu, W. & Cherezov, V. (2015). *IUCrJ*, **2**, 545–551. 10.1107/S2052252515013160 PMC4547822 26306196

[bb28] Garman, E. F. (2010). *Acta Cryst.* D**66**, 339–351. 10.1107/S0907444910008656 PMC2852297 20382986

[bb29] Garman, E. F. & McSweeney, S. M. (2007). *J. Synchrotron Rad.* **14**, 1–3. 10.1107/S0909049506053015 17211066

[bb30] Gati, C., Bourenkov, G., Klinge, M., Rehders, D., Stellato, F., Oberthür, D., Yefanov, O., Sommer, B. P., Mogk, S., Duszenko, M., Betzel, C., Schneider, T. R., Chapman, H. N. & Redecke, L. (2014). *IUCrJ*, **1**, 87–94. 10.1107/S2052252513033939 PMC4062088 25075324

[bb31] Genick, U. K., Borgstahl, G. E. O., Ng, K., Ren, Z., Pradervand, C., Burke, P. M., Srajer, V., Teng, T.-Y., Schildkamp, W., McRee, D. E., Moffat, K. & Getzoff, E. D. (1997). *Science*, **275**, 1471–1475. 10.1126/science.275.5305.1471 9045611

[bb32] Hajdu, J. & Johnson, L. N. (1990). *Biochemistry*, **29**, 1669–1678. 10.1021/bi00459a001 2184884

[bb33] Hammouda, B., Ho, D. L. & Kline, S. (2004). *Macromolecules*, **37**, 6932–6937.

[bb34] Hasegawa, K., Yamashita, K., Murai, T., Nuemket, N., Hirata, K., Ueno, G., Ago, H., Nakatsu, T., Kumasaka, T. & Yamamoto, M. (2017). *J. Synchrotron Rad.* **24**, 29–41. 10.1107/S1600577516016362 PMC5182019 28009544

[bb35] Hau-Riege, S. P. & Bennion, B. J. (2015). *Phys. Rev. E*, **91**, 022705. 10.1103/PhysRevE.91.022705 25768529

[bb36] Heymann, M., Opthalage, A., Wierman, J. L., Akella, S., Szebenyi, D. M. E., Gruner, S. M. & Fraden, S. (2014). *IUCrJ*, **1**, 349–360. 10.1107/S2052252514016960 PMC4174877 25295176

[bb37] Hilgart, M. C., Sanishvili, R., Ogata, C. M., Becker, M., Venugopalan, N., Stepanov, S., Makarov, O., Smith, J. L. & Fischetti, R. F. (2011). *J. Synchrotron Rad.* **18**, 717–722. 10.1107/S0909049511029918 PMC3161817 21862850

[bb38] Huang, C.-Y., Olieric, V., Ma, P., Howe, N., Vogeley, L., Liu, X., Warshamanage, R., Weinert, T., Panepucci, E., Kobilka, B., Diederichs, K., Wang, M. & Caffrey, M. (2016). *Acta Cryst.* D**72**, 93–112. 10.1107/S2059798315021683 PMC4756617 26894538

[bb39] Huang, C.-Y., Olieric, V., Ma, P., Panepucci, E., Diederichs, K., Wang, M. & Caffrey, M. (2015). *Acta Cryst.* D**71**, 1238–1256. 10.1107/S1399004715005210 PMC4461204 26057665

[bb40] Kabsch, W. (2010). *Acta Cryst.* D**66**, 125–132. 10.1107/S0907444909047337 PMC2815665 20124692

[bb41] Katritch, V., Fenalti, G., Abola, E. E., Roth, B. L., Cherezov, V. & Stevens, R. C. (2014). *Trends Biochem. Sci.* **39**, 233–244. 10.1016/j.tibs.2014.03.002 PMC4106411 24767681

[bb42] Keedy, D. A., van den Bedem, H., Sivak, D. A., Petsko, G. A., Ringe, D., Wilson, M. A. & Fraser, J. S. (2014). *Structure*, **22**, 899–910. 10.1016/j.str.2014.04.016 PMC4082491 24882744

[bb43] Kirian, R. A., Wang, X., Weierstall, U., Schmidt, K. E., Spence, J. C. H., Hunter, M., Fromme, P., White, T., Chapman, H. N. & Holton, J. (2010). *Opt. Express*, **18**, 5713–5723. 10.1364/OE.18.005713 PMC4038330 20389587

[bb44] Kirian, R. A., White, T. A., Holton, J. M., Chapman, H. N., Fromme, P., Barty, A., Lomb, L., Aquila, A., Maia, F. R. N. C., Martin, A. V., Fromme, R., Wang, X., Hunter, M. S., Schmidt, K. E. & Spence, J. C. H. (2011). *Acta Cryst.* A**67**, 131–140. 10.1107/S0108767310050981 PMC3066792 21325716

[bb45] Kissick, D. J., Wanapun, D. & Simpson, G. J. (2011). *Annu. Rev. Anal. Chem.* **4**, 419–437. 10.1146/annurev.anchem.111808.073722 PMC3345893 21469954

[bb46] Kupitz, C., Grotjohann, I., Conrad, C. E., Roy-Chowdhury, S., Fromme, R. & Fromme, P. (2014). *Philos. Trans. R. Soc. Lond B Biol. Sci.* **369**, 20130316. 10.1098/rstb.2013.0316 PMC4052858 24914149

[bb47] Liu, W., Chun, E., Thompson, A. A., Chubukov, P., Xu, F., Katritch, V., Han, G. W., Roth, C. B., Heitman, L. H., IJzerman, A. P., Cherezov, V. & Stevens, R. C. (2012). *Science*, **337**, 232–236. 10.1126/science.1219218 PMC3399762 22798613

[bb48] Liu, W., Ishchenko, A. & Cherezov, V. (2014). *Nat. Protoc.* **9**, 2123–2134. 10.1038/nprot.2014.141 PMC4209290 25122522

[bb49] Lomb, L. *et al.* (2011). *Phys. Rev. B*, **84**, 214111. 10.1103/PhysRevB.84.214111 PMC3786679 24089594

[bb50] Low, B. W., Chen, C. C. H., Berger, J. E., Singman, L. & Pletcher, J. F. (1966). *Proc. Natl Acad. Sci. USA*, **56**, 1746–1750. 10.1073/pnas.56.6.1746 PMC220166 16591415

[bb51] Macchi, P. (2012). *Top. Curr. Chem.* **315**, 33–67. 10.1007/128_2011_207 21928014

[bb52] Marín, M., Thallmair, V. & Ott, T. (2012). *J. Biol. Chem.* **287**, 39982–39991. 10.1074/jbc.M112.414292 PMC3501056 23027878

[bb53] Martin-Garcia, J. M., Conrad, C. E., Coe, J., Roy-Chowdhury, S. & Fromme, P. (2016). *Arch. Biochem. Biophys.* **602**, 32–47. 10.1016/j.abb.2016.03.036 PMC4909539 27143509

[bb54] Martin-Garcia, J. M., Hansen, D. T., Zook, J., Loskutov, A. V., Robida, M. D., Craciunescu, F. M., Sykes, K. F., Wachter, R. M., Fromme, P. & Allen, J. P. (2014). *Biochemistry*, **53**, 1958–1970. 10.1021/bi401644s PMC3985703 24593131

[bb55] McCoy, A. J., Grosse-Kunstleve, R. W., Adams, P. D., Winn, M. D., Storoni, L. C. & Read, R. J. (2007). *J. Appl. Cryst.* **40**, 658–674. 10.1107/S0021889807021206 PMC2483472 19461840

[bb56] Murray, T. D., Lyubimov, A. Y., Ogata, C. M., Vo, H., Uervirojnangkoorn, M., Brunger, A. T. & Berger, J. M. (2015). *Acta Cryst.* D**71**, 1987–1997. 10.1107/S1399004715015011 PMC4601365 26457423

[bb57] Murshudov, G. N., Skubák, P., Lebedev, A. A., Pannu, N. S., Steiner, R. A., Nicholls, R. A., Winn, M. D., Long, F. & Vagin, A. A. (2011). *Acta Cryst.* D**67**, 355–367. 10.1107/S0907444911001314 PMC3069751 21460454

[bb58] Nave, C. & Garman, E. F. (2005). *J. Synchrotron Rad.* **12**, 257–260. 10.1107/S0909049505007132 15840908

[bb59] Neutze, R., Wouts, R., van der Spoel, D., Weckert, E. & Hajdu, J. (2000). *Nature (London)*, **406**, 752–757. 10.1038/35021099 10963603

[bb60] Nogly, P. *et al.* (2015). *IUCrJ*, **2**, 168–176. 10.1107/S2052252514026487 PMC4392771 25866654

[bb61] Nyarko, A., Hare, M., Hays, T. S. & Barbar, E. (2004). *Biochemistry*, **43**, 15595–15603. 10.1021/bi048451+ 15581372

[bb62] Owen, R. L., Axford, D., Nettleship, J. E., Owens, R. J., Robinson, J. I., Morgan, A. W., Doré, A. S., Lebon, G., Tate, C. G., Fry, E. E., Ren, J., Stuart, D. I. & Evans, G. (2012). *Acta Cryst.* D**68**, 810–818. 10.1107/S0907444912012553 PMC4791751 22751666

[bb63] Pflugrath, J. W. (2004). *Methods*, **34**, 415–423. 10.1016/j.ymeth.2004.03.032 15325658

[bb64] Pietras, Z., Lin, H.-T., Surade, S., Luisi, B., Slattery, O., Pos, K. M. & Moreno, A. (2010). *J. Appl. Cryst.* **43**, 58–63.

[bb65] Pothineni, S. B., Venugopalan, N., Ogata, C. M., Hilgart, M. C., Stepanov, S., Sanishvili, R., Becker, M., Winter, G., Sauter, N. K., Smith, J. L. & Fischetti, R. F. (2014). *J. Appl. Cryst.* **47**, 1992–1999. 10.1107/S1600576714022730 PMC4248568 25484844

[bb66] Powell, H. R., Johnson, O. & Leslie, A. G. W. (2013). *Acta Cryst.* D**69**, 1195–1203. 10.1107/S0907444912048524 PMC3689522 23793145

[bb67] Ravelli, R. B. G. & Garman, E. F. (2006). *Curr. Opin. Struct. Biol.* **16**, 624–629. 10.1016/j.sbi.2006.08.001 16938450

[bb68] Redecke, L. *et al.* (2013). *Science*, **339**, 227–230.

[bb69] Sanishvili, R., Nagarajan, V., Yoder, D., Becker, M., Xu, S., Corcoran, S., Akey, D. L., Smith, J. L. & Fischetti, R. F. (2008). *Acta Cryst.* D**64**, 425–435. 10.1107/S0907444908001741 PMC2631116 18391409

[bb70] Sauter, N. K. (2015). *J. Synchrotron Rad.* **22**, 239–248. 10.1107/S1600577514028203 PMC4344359 25723925

[bb71] Sawaya, M. R. *et al.* (2014). *Proc. Natl Acad. Sci. USA*, **111**, 12769–12774.

[bb72] Schirmer, T., Bode, W., Huber, R., Sidler, W. & Zuber, H. (1985). *J. Mol. Biol.* **184**, 257–277. 10.1016/0022-2836(85)90379-1 3928897

[bb73] Schotte, F., Cho, H. S., Kaila, V. R., Kamikubo, H., Dashdorj, N., Henry, E. R., Graber, T. J., Henning, R., Wulff, M., Hummer, G., Kataoka, M. & Anfinrud, P. A. (2012). *Proc. Natl Acad. Sci. USA*, **109**, 19256–19261. 10.1073/pnas.1210938109 PMC3511082 23132943

[bb75] Sierra, R. G. *et al.* (2012). *Acta Cryst.* D**68**, 1584–1587.

[bb76] Stellato, F. *et al.* (2014). *IUCrJ*, **1**, 204–212. 10.1107/S2052252514010070 PMC4107920 25075341

[bb77] Stepanov, S., Makarov, O., Hilgart, M., Pothineni, S. B., Urakhchin, A., Devarapalli, S., Yoder, D., Becker, M., Ogata, C., Sanishvili, R., Venugopalan, N., Smith, J. L. & Fischetti, R. F. (2011). *Acta Cryst.* D**67**, 176–188. 10.1107/S0907444910053916 PMC3046456 21358048

[bb78] Su, J., Yang, J., Zhao, D., Kawula, T. H., Banas, J. A. & Zhang, J. R. (2007). *Infect. Immun.* **75**, 3089–3101. 10.1128/IAI.01865-06 PMC1932872 17420240

[bb79] Terwilliger, T. C., Dimaio, F., Read, R. J., Baker, D., Bunkóczi, G., Adams, P. D., Grosse-Kunstleve, R. W., Afonine, P. V. & Echols, N. (2012). *J. Struct. Funct. Genomics*, **13**, 81–90. 10.1007/s10969-012-9129-3 PMC3375004 22418934

[bb80] Terwilliger, T. C., Grosse-Kunstleve, R. W., Afonine, P. V., Moriarty, N. W., Zwart, P. H., Hung, L.-W., Read, R. J. & Adams, P. D. (2008). *Acta Cryst.* D**64**, 61–69. 10.1107/S090744490705024X PMC2394820 18094468

[bb81] Vagin, A. & Teplyakov, A. (2010). *Acta Cryst.* D**66**, 22–25. 10.1107/S0907444909042589 20057045

[bb82] Warkentin, M., Badeau, R., Hopkins, J. & Thorne, R. E. (2011). *Acta Cryst.* D**67**, 792–803. 10.1107/S0907444911027600 PMC3169314 21904032

[bb83] Warkentin, M., Hopkins, J. B., Badeau, R., Mulichak, A. M., Keefe, L. J. & Thorne, R. E. (2013). *J. Synchrotron Rad.* **20**, 7–13. 10.1107/S0909049512048303 PMC3526918 23254651

[bb84] Weierstall, U. *et al.* (2014). *Nat. Commun.* **5**, 3309. 10.1038/ncomms4309 PMC4061911 24525480

[bb85] Weierstall, U., Spence, J. C. H. & Doak, R. B. (2012). *Rev. Sci. Instrum.* **83**, 035108. 10.1063/1.3693040 22462961

[bb86] White, T. A., Barty, A., Stellato, F., Holton, J. M., Kirian, R. A., Zatsepin, N. A. & Chapman, H. N. (2013). *Acta Cryst.* D**69**, 1231–1240. 10.1107/S0907444913013620 PMC3689526 23793149

[bb87] White, T. A., Kirian, R. A., Martin, A. V., Aquila, A., Nass, K., Barty, A. & Chapman, H. N. (2012). *J. Appl. Cryst.* **45**, 335–341.

[bb88] White, T. A., Mariani, V., Brehm, W., Yefanov, O., Barty, A., Beyerlein, K. R., Chervinskii, F., Galli, L., Gati, C., Nakane, T., Tolstikova, A., Yamashita, K., Yoon, C. H., Diederichs, K. & Chapman, H. N. (2016). *J. Appl. Cryst.* **49**, 680–689. 10.1107/S1600576716004751 PMC4815879 27047311

[bb89] Xiang, J., Chun, E., Liu, C., Jing, L., Al-Sahouri, Z., Zhu, L. & Liu, W. (2016). *Trends Pharmacol. Sci.* **37**, 1055–1069. 10.1016/j.tips.2016.09.009 27726881

[bb90] Yazawa, K., Sugahara, M., Yutani, K., Takehira, M. & Numata, K. (2016). *ACS Catal.* **6**, 3036–3046.

[bb91] Zander, U., Bourenkov, G., Popov, A. N., de Sanctis, D., Svensson, O., McCarthy, A. A., Round, E., Gordeliy, V., Mueller-Dieckmann, C. & Leonard, G. A. (2015). *Acta Cryst.* D**71**, 2328–2343. 10.1107/S1399004715017927 PMC4631482 26527148

[bb92] Zeldin, O. B., Brockhauser, S., Bremridge, J., Holton, J. M. & Garman, E. F. (2013). *Proc. Natl Acad. Sci. USA*, **110**, 20551–20556. 10.1073/pnas.1315879110 PMC3870734 24297937

[bb93] Zeldin, O. B., Gerstel, M. & Garman, E. F. (2013). *J. Synchrotron Rad.* **20**, 49–57. 10.1107/S0909049512044706 23254655

[bb94] Zook, J., Mo, G., Sisco, N. J., Craciunescu, F. M., Hansen, D. T., Baravati, B., Cherry, B. R., Sykes, K., Wachter, R., Van Horn, W. D. & Fromme, P. (2015). *Structure*, **23**, 1116–1122. 10.1016/j.str.2015.03.025 PMC4835214 26004443

