# Supplementary material for: Serial millisecond crystallography of membrane and soluble protein microcrystals using synchrotron radiation
Source: IUCrJ. 2017 May 24;4(Pt 4):439–54. doi: 10.1107/S205225251700570X (PMC5571807; doi:10.1107/S205225251700570X)
Supplement: Supplementary file 1 [file m-04-00439-sup1.pdf]

IUCrJ

Volume 4 (2017)

Supporting information for article:

Serial millisecond crystallography of membrane and soluble protein microcrystals using synchrotron radiation

Jose Manuel Martin-Garcia, Chelsie E. Conrad, Garrett Nelson, Natasha Stander, Nadia A. Zatsepin, James Zook, Lan Zhu, James Geiger, Eugene Chun, David Kissick, Mark C. Hilgart, Craig Ogata, Andrii Ishchenko, Nirupa Nagaratnam, Shatabdi Roy-Chowdhury, Jesse Coe, Ganesh Subramanian, Alexander Schaffer, Daniel James, Gihan Ketwala, Nagarajan Venugopalan, Shenglan Xu, Stephen Corcoran, Dale Ferguson, Uwe Weierstall, John C. H. Spence, Vadim Cherezov, Petra Fromme, Robert F. Fischetti and Wei Liu

S1. Diffuse background X-ray scattering comparison

We compared the diffuse background X-ray scattering from 50 µm-diameter streams of PEO gel versus that from LCP using detector readout frames with no more than 2 peaks found by Cheetah-cbf. Frames that contained no scattering from the stream, due to the jet temporarily fluctuating out of the X-ray path, were excluded from the analysis by their low photon counts (average radial intensity of < 1 photon). The total number of frames containing scattering from the stream alone were 24,234 for lysozyme in LCP and 26,946 for PC in PEO gel.

.

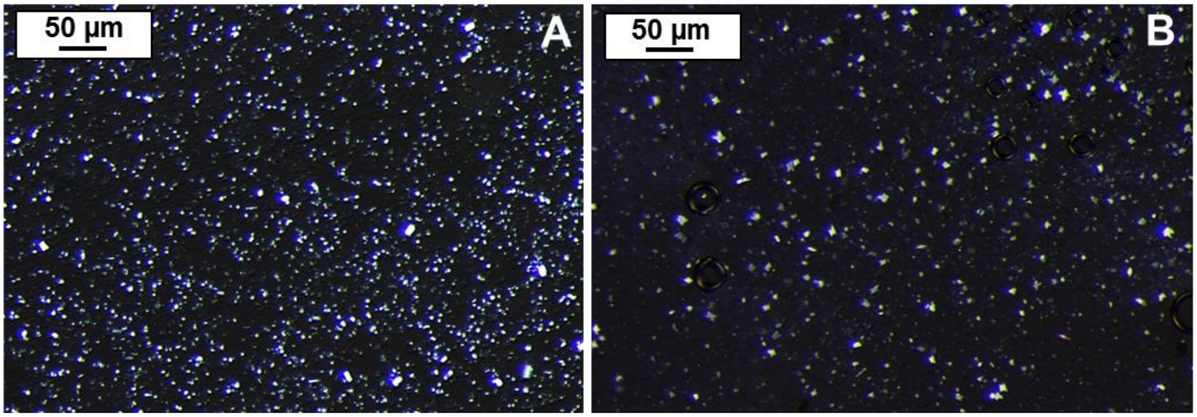

Figure S1 Cross-polarized images of micro-crystals of $A_{2A}AR$ (A) and lysozyme (B) mixed with LCP

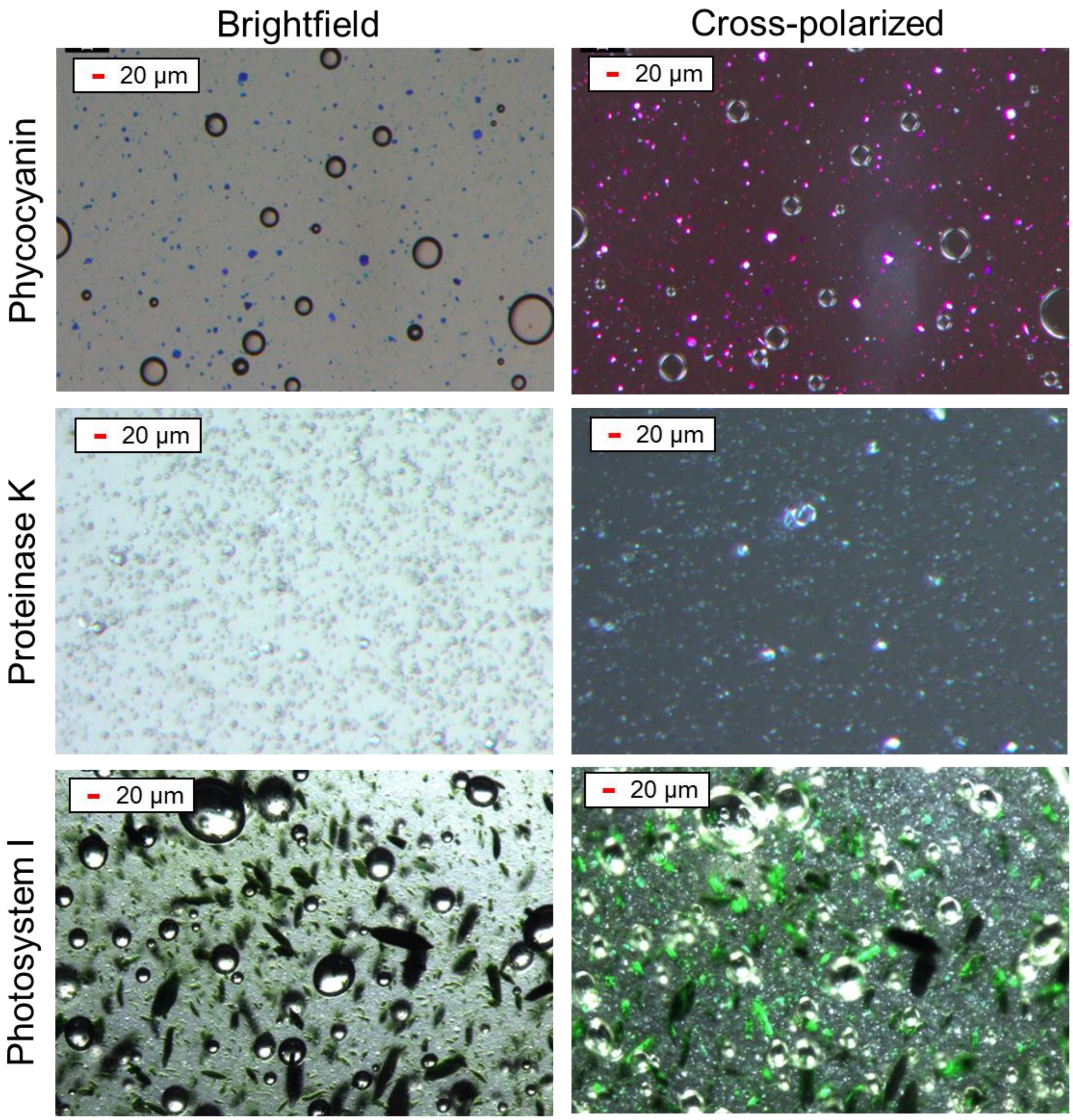

Figure S2 Bright field (left column) and cross-polarized (right column) images of micro-crystals of phycocyanin, proteinase K, and photosystem I embedded into the PEO gel.

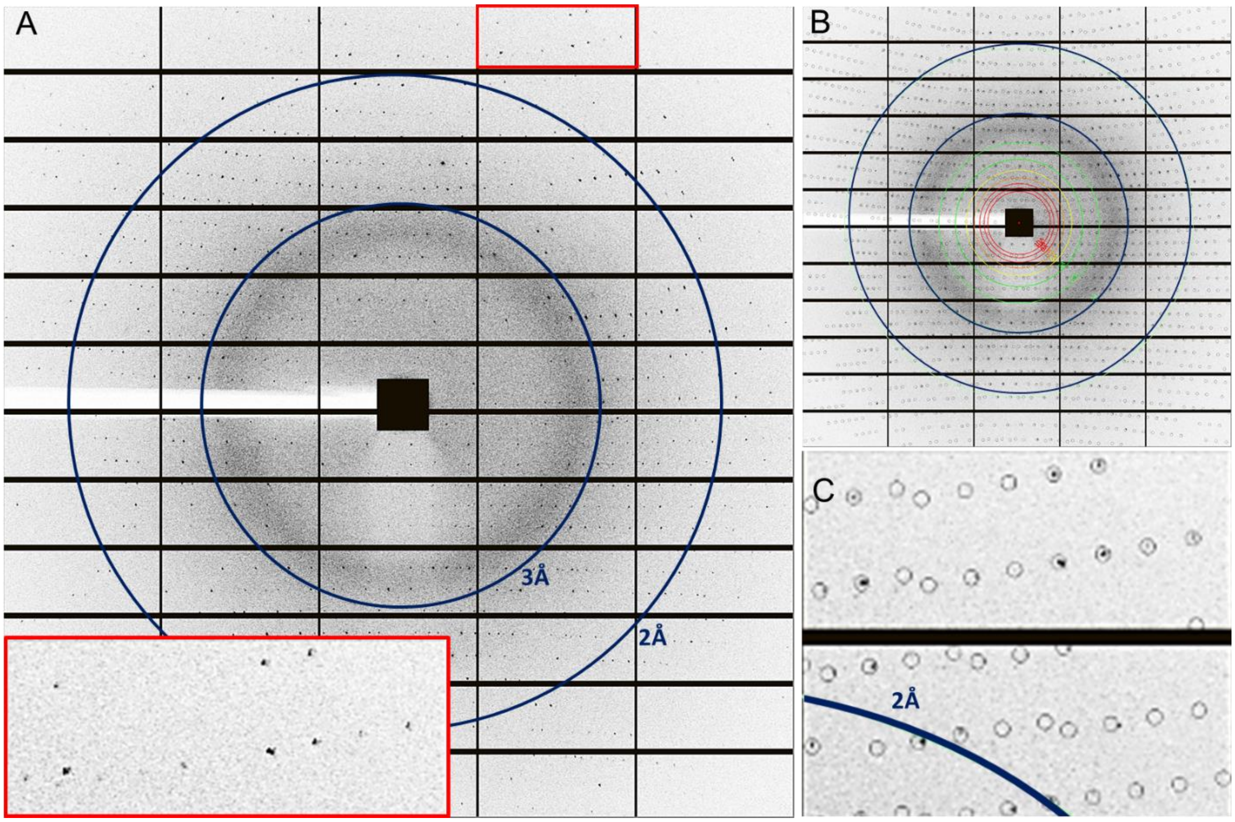

Figure S3 Diffraction pattern from a single lysozyme micro-crystal in LCP. A) Diffraction pattern showing Bragg peaks visible beyond 2 Å resolution (red boxed inlet panel). B) Same diffraction pattern in A) after indexing with resolution rings. C) Closer view of the red boxed area highlighted in B).

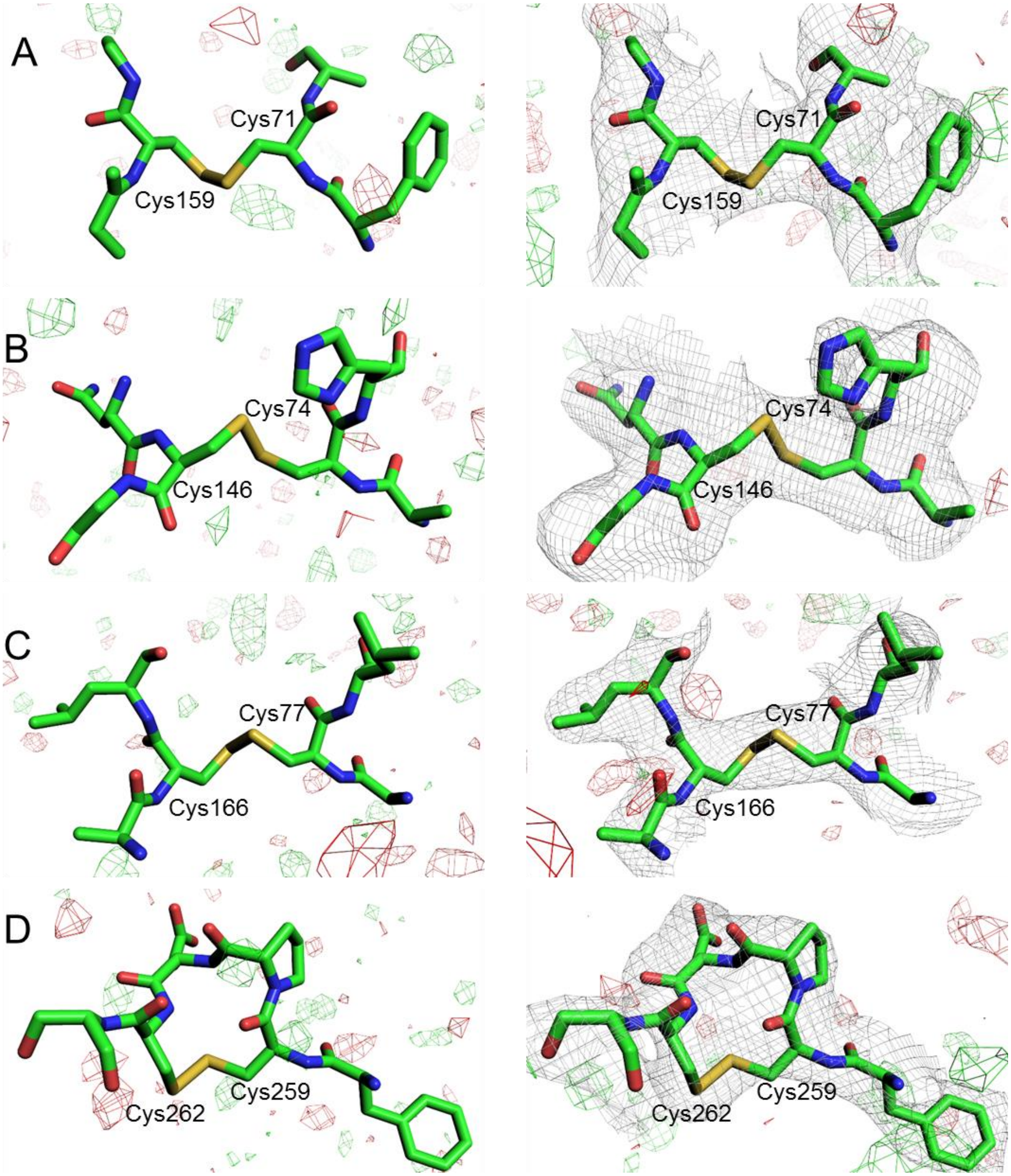

Figure S4 Electron density maps displayed around the four disulfide bridges of $A_{2A}AR$ (Cys71-Cys159 (A), Cys74-Cys146 (B), Cys77-Cys166 (C), Cys259-Cys262 (D)). Cysteines and neighboring residues are shown as green stick representation. Left panels: Structure factor amplitude Fourier difference (Fo-Fo) maps at 3σ between our data set and the data set collected at LCLS (Batyuk *et al.*, 2016), with red and

green contours indicate negative and positive density, respectively. Right panels: 2mFo-DFc (light grey mesh, contour at 1σ) and mFo-Fc (red and green meshes, contour at 3σ) maps.

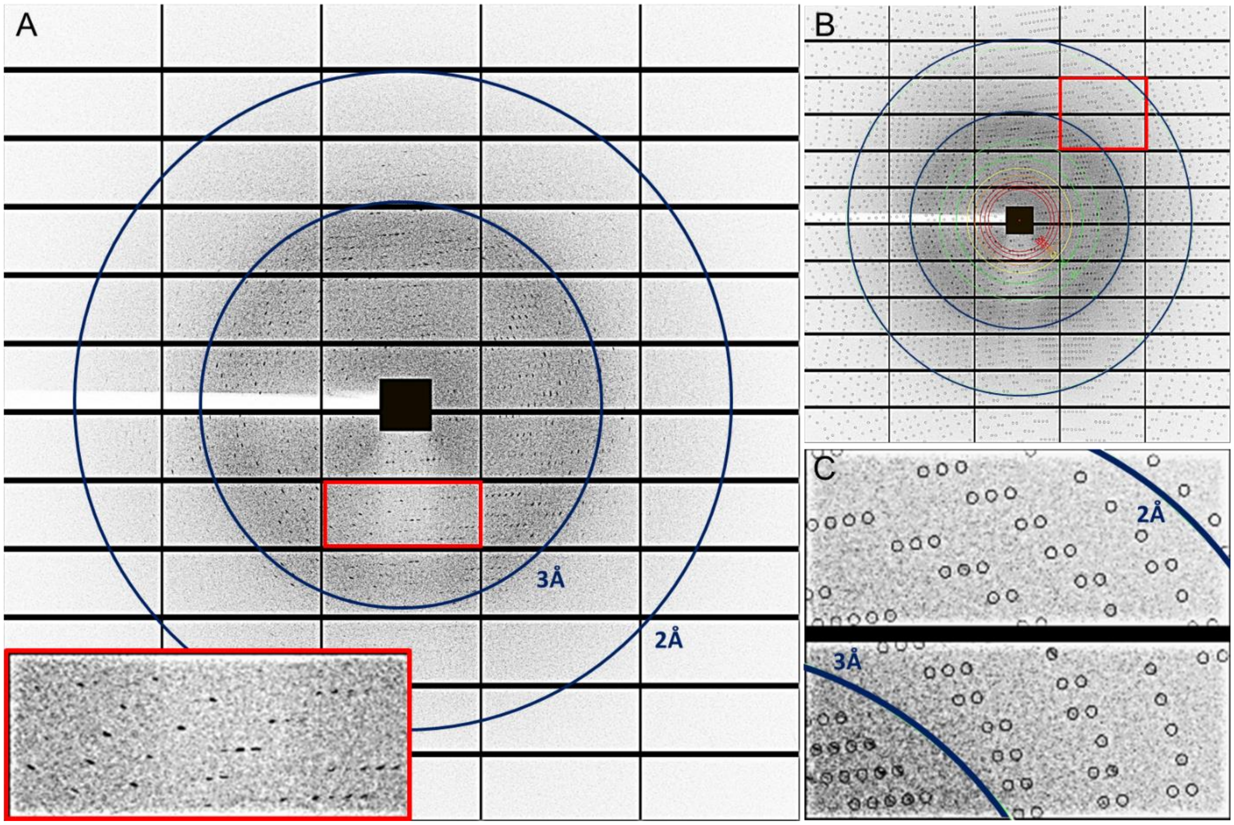

Figure S5 Diffraction pattern of a single micro-crystal of PC in PEO. A) Diffraction pattern with resolution rings at 2 Å and 3 Å resolution shows visible Bragg spots extending out to about 2.2 Å. Red boxed inlet shows smeared Bragg spots due to crystal rotation during the 100 msec exposure time. B) Same diffraction pattern in A) after indexing with resolution rings. C) Closer view of the red boxed area highlighted in B).

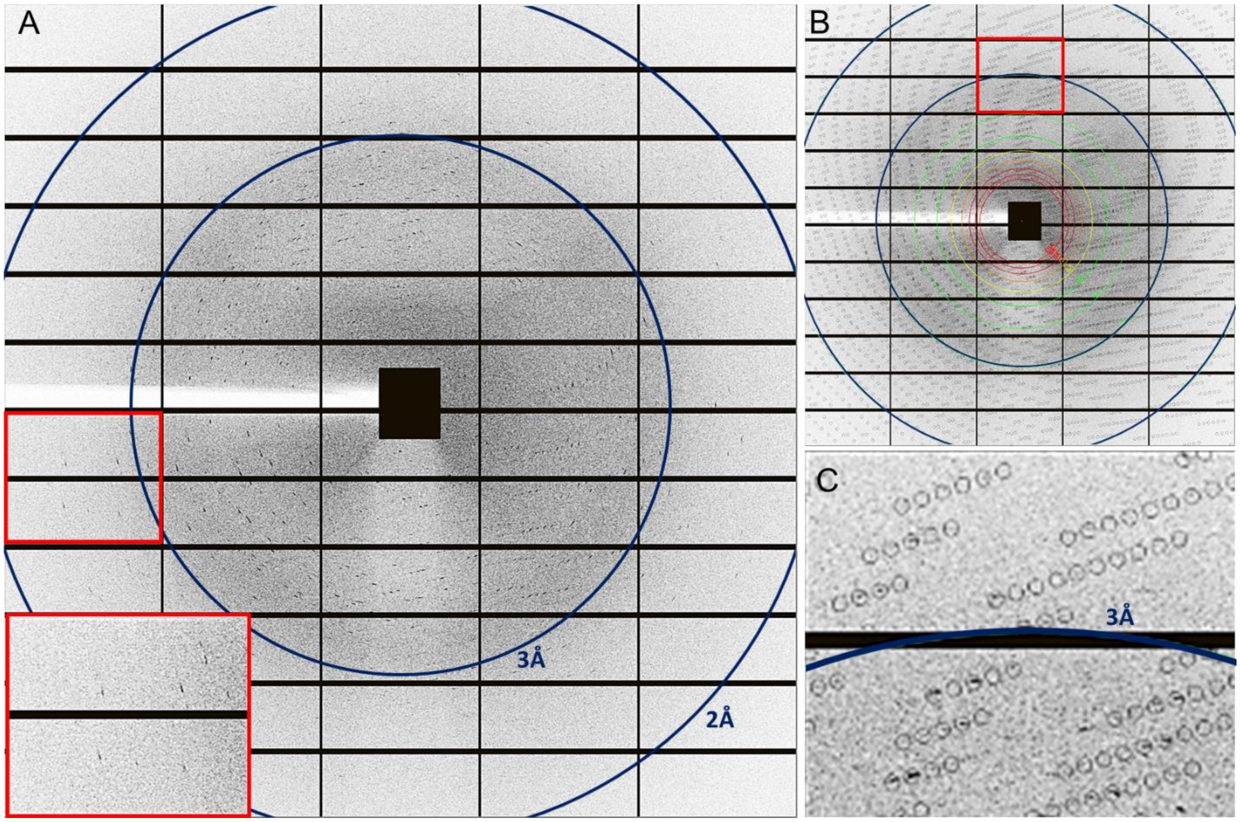

Figure S6 Diffraction pattern of a single micro-crystal of proteinase K in PEO. A) Diffraction pattern with resolution rings at 2 Å and 3 Å resolution shows visible Bragg spots extending out to about 2.3 Å (red boxed inlet panel). Red boxed inlet also shows smeared Bragg spots due to crystal rotation during the 100 msec exposure time. B) Same diffraction pattern in A) after indexing with resolution rings. C) Closer view of the red boxed area highlighted in B).

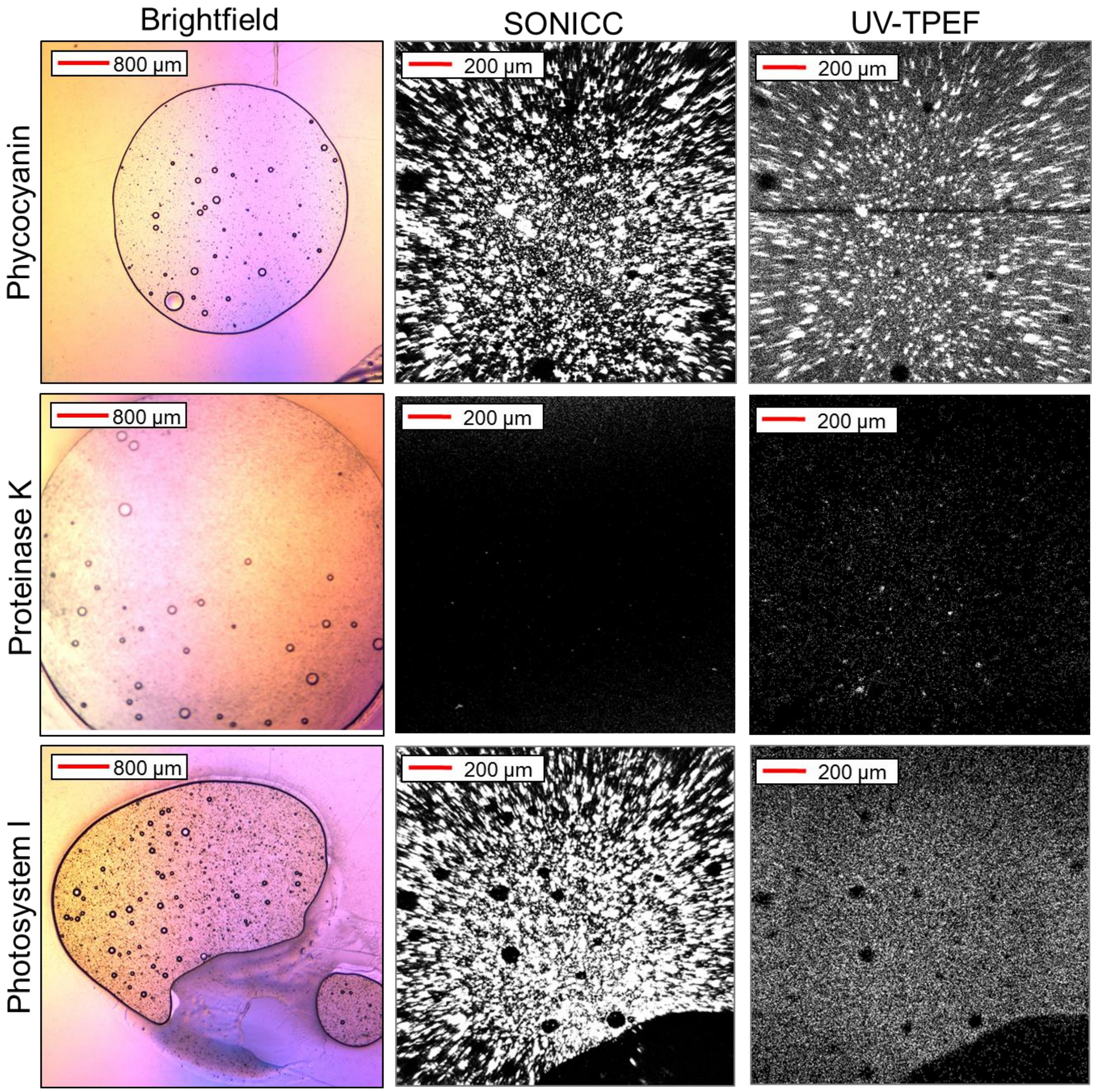

Figure S7 Bright field, SONICC and UV-TPEF images of phycocyanin, proteinase K and photosystem I micro-crystals mixed with PEO gel

Reference

Batyuk, A., Galli, L., Ishchenko, A., Han, G. W., Gati, C., Popov, P. A., Lee, M. Y., Stauch, B., White, T. A., Barty, A., Aquila, A., Hunter, M. S., Liang, M., Boutet, S., Pu, M., Liu, Z. J., Nelson, G., James, D., Li, C., Zhao, Y., Spence, J. C., Liu, W., Fromme, P., Katritch, V., Weierstall, U., Stevens, R. C. & Cherezov, V. (2016). *Sci Adv* **2**, e1600292.
